# Effects of air pollution on restricted activity days: systematic review and meta-analysis

**DOI:** 10.1186/s12940-023-00979-8

**Published:** 2023-03-30

**Authors:** Pablo Orellano, Julieta Reynoso, Nancy Quaranta

**Affiliations:** 1 Consejo Nacional de Investigaciones Científicas y Técnicas (CONICET), Buenos Aires, Argentina; 2 Universidad Tecnológica Nacional, Facultad Regional San Nicolás, Colon 332, San Nicolas de los Arroyos, Argentina; 3 Hospital General “San Felipe”, San Nicolás, Argentina; 4 Comisión de Investigaciones Científicas, Provincia de Buenos Aires, La Plata, Argentina

**Keywords:** Air pollution, Absenteeism, Observational study, Systematic review, Meta-analysis

## Abstract

**Background:**

The adverse effects of air pollution on human health include many diseases and health conditions associated with mortality, morbidity and disability. One example of these outcomes that can be translated into economic costs is the number of days of restricted activity. The aim of this study was to assess the effect of outdoor exposure to particulate matter with an aerodynamic diameter less than or equal to 10 and 2.5 μm (PM_10_, PM_2.5_), nitrogen dioxide (NO_2_), and ozone (O_3_), on restricted activity days.

**Methods:**

Observational epidemiological studies with different study designs were included, and pooled relative risks (RR) with 95% confidence intervals (95%CI) were calculated for an increase of 10 μg/m^3^ of the pollutant of interest. Random-effects models were chosen because of the environmental differences between the studies. Heterogeneity was estimated using prediction intervals (PI) and I-Squared (I2) values, while risk of bias was assessed using a tool developed by the World Health Organization specifically designed for air pollution studies, and based on different domains. Subgroup and sensitivity analyses were performed where possible. The protocol for this review was registered with PROSPERO (CRD42022339607).

**Results:**

We included 18 articles in the quantitative analysis. Associations between pollutants and restricted activity days in time-series studies of short-term exposures, measured as work-loss days, school-loss days, or both were significant for PM_10_ (RR: 1.0191; 95%CI: 1.0058–1.0326; 80%PI: 0.9979–1.0408; I2: 71%) and PM_2.5_ (RR: 1.0166; 95%CI: 1.0050–1.0283; 80%PI: 0.9944–1.0397; I2: 99%), but not for NO_2_ or O_3_. Some degree of heterogeneity between studies was observed, but sensitivity analysis showed no differences in the direction of the pooled relative risks when studies with a high risk of bias were excluded. Cross-sectional studies also showed significant associations for PM_2.5_ and restricted activity days. We could not perform the analysis for long-term exposures because only two studies analysed this type of association.

**Conclusion:**

Restricted activity days and related outcomes were associated with some of the pollutants under evaluation, as shown in studies with different designs. In some cases, we were able to calculate pooled relative risks that can be used for quantitative modelling.

**Supplementary Information:**

The online version contains supplementary material available at 10.1186/s12940-023-00979-8.

## Background

Over time, more and more evidence has been published on the adverse health effects of air pollution on human health, even at low concentrations of air pollutants [1]. In 2021, the new update of the World Health Organization (WHO) Air Quality Guidelines collected and reported new evidence on the health effect associated with exposure to ambient air pollution [2]. The WHO document also highlighted the fact that reducing air pollution levels would bring health benefits, even in places with low levels of pollution [3]. Exposure to air pollution is known to increase the risk of several non-communicable cardiovascular and respiratory diseases, lower respiratory tract infections, and other health conditions [2], while many others are under investigation. However, morbidity and mortality are not the only evidence of the link between air pollution and health; reducing air pollution concentrations to fit the levels recommended in the WHO Air Quality Guidelines would bring additional benefits by reducing social inequalities and the economic costs associated with the health effects of air pollution exposure [4]. Air pollution causes significant losses in productivity and social well-being, mainly through reduced life expectancy [5] and quality of life. On the other hand, air pollution affects labour productivity because workers may take more sick days (absenteeism) or work less productively [6], the latter effect also known as "presenteeism" [7]. Another type of absenteeism that is often associated with exposure to air pollution, but which affects different age groups, is school absenteeism. School absenteeism is usually related to illness or injury, although it is not always caused by chronic or other diseases [8]. The association between school absenteeism and exposure to air pollution has been documented in epidemiological studies, as has the association with work absenteeism.

Work and school absenteeism, together with days spent in bed due to illness, and any other form of restricted activity, can be included in the broad concept of restricted activity days. In this way, a restricted activity day has been defined as a day on which a person reduces his or her normal activities for the whole day because of illness or injury; bed days, school-loss days (school absenteeism), and work-loss days (work absenteeism) are included in the total number of restricted-activity days [9]. Minor restricted activity days are defined as days on which most usual daily activities are reduced, but without falling into school or work absenteeism [10]. These days with minor restrictions are also included in the measure of restricted activity days [11], but the economic costs associated with restricted activity days and minor restricted activity days are different. These estimates are important components of health impact assessments and predictive models for assessing the economic impacts of ambient air pollution. To include these associations as relevant parameters in predictive models, valid relative risks and concentration–response functions should be obtained from observational epidemiological studies. However, despite the importance of restricted activity days as a direct consequence of air pollution exposure, the number of studies assessing these associations is relatively small and estimates are generally based on studies published in the 1990s. WHO is currently coordinating a project entitled “Estimation of Morbidity from Air Pollution and its Economic Costs” (EMAPEC), which aims to establish a methodology for estimating the economic costs of selected morbidity outcomes arising from the exposure to air pollution, and to test its application at different geographical scales (national, regional and global). This project requires different concentration–response functions and concentration-effect estimates are needed between exposure to air pollution and different outcomes indicating direct and indirect costs. Among these outcomes, restricted activity days are relevant endpoints to be considered in predictive models. In this context, this systematic review and meta-analysis was commissioned by WHO to assess the association between outdoor exposure to common air pollutants, including particulate matter with an aerodynamic diameter of 10 and 2.5 μm (PM_10_ and PM_2.5_), nitrogen dioxide (NO_2_) and ozone (O_3_), and restricted activity days.

## Methods

### Protocol and registration

The protocol for this systematic review was developed prior to the formal search for articles, and registered with PROSPERO (http://www.crd.york.ac.uk/PROSPERO/) under registration number CRD42022339607.

### Research question

The research question for this systematic review was formulated as a Population, Exposure, Comparator, Outcome and Study design (PECOS) question, as elaborated by Morgan and colleagues [12]:

In any population, including subgroups of susceptible adults and children (P), what is the effect of the exposure to ambient concentrations of PM_2.5_, PM_10_, O_3_, and NO_2_ (E), versus the exposure to lower levels of air pollution (C) (difference of 10 μg/m^3^), on the number of restricted activity days (O), as observed in observational epidemiological studies (S)?

### Search strategy

The search included terms related to the exposure (pollutants) and the outcomes, taking into account synonyms, symbols, formulae and abbreviations. A detail of this search strategy applied to one specific database is shown in Supplementary Table 1, Additional file 1. The database search included the following sources: Medline via PubMed, Scopus via Elsevier, and a series of regional databases including Literatura Latinoamericana y del Caribe en Ciencias de la Salud (LILACS), Western Pacific Region Index Medicus (WPRIM), Index Medicus for South-East Asia Region (IMSEAR), Index Medicus for the Eastern Mediterranean Region (IMEMR), and African Index Medicus (AIM). These regional databases are potential sources of peer-reviewed scientific articles, grey literature, and journals from developing countries, improving the search and enabling the inclusion of local reports. Additional articles found in the reference lists of selected reviews and guidelines, as well as articles identified with the help of experts, were also included. The search included studies up to June 2022, with no language restrictions. The abstracts and full-text articles selected for inclusion that were written in a language other than English were translated using an online translation tool (Google Translate).

Screening of titles, abstracts and full texts was carried out by two reviewers (PO and JR), and in case of disagreement, a third reviewer (NQ) made the final decision on inclusion. Each stage of this process was recorded in spreadsheets developed in Excel®.

### Eligibility process

The independent variable of interest was the short- or long-term exposure to ambient concentrations of PM_10_, PM_2.5_, NO_2_ and O_3_, irrespective of the pollution source, and expressed in a concentration unit (e.g. μg/m^3^, ppb). The comparison was made with the same or with a control population exposed to a lower level of air pollutants, taking into account a standardised difference in the concentration (10 μg/m^3^).

The outcomes of interest were days with restricted activity or restricted activity days, which may have included bed-days due to morbidity, school-loss days, work-loss days, and minor restricted activity days. All of these outcomes were often related to respiratory-illnesses and other diseases and conditions, but in some studies, particularly for school-loss days, absenteeism from all causes was included. However, when the same article reported both outcomes, i.e. all-causes absenteeism and illness-related absenteeism, the latter was selected for inclusion, because it was considered to be more related to the effects of air pollution.

For this review we included all observational studies following epidemiological designs including cohort, panel, case-crossover, ecological time-series, and cross-sectional studies. Interventional studies, physiological studies and animal studies were excluded. Studies dealing only with indoor or occupational exposures, qualitative or modelling studies, reviews and guidelines were also excluded. We did not restrict our inclusion criteria to a specific time-lag between the exposure and the outcome; typically this is shorter in time-series studies than in cross-sectional or cohort designs. However, we performed different analyses for different types of study design. When multiple lags between the exposure and the outcome were reported in the same study, as is often the case in time-series studies, we followed the algorithm developed by Atkinson and colleagues [13], as shown below:

- (1) The lag that the author focused on or specified a priori.
- (2) the most statistically significant lag (positive or negative).
- (3) The lag with the largest effect estimate (positive or negative).

With regard to modelling strategies and association measures, we considered studies eligible if they reported relative risks (RR), odds ratios (OR), percent excess risks (ER%), hazard ratios (HR), or regression model coefficients. To ensure comparability between studies and effect sizes, only studies using generalised linear and generalised additive models were included in the analysis.

If there was a complete or partial overlap of data in two or more articles, the article was selected for inclusion according to the following criteria and in the following order: 1) wider geographical distribution; 2) longer duration of the study period; and 3) more recent publication date.

### Data extraction and procedures

Two independent reviewers (PO and JR) independently screened the titles and abstracts identified by the systematic search, and in a later step the same reviewers extracted the information from selected articles. Disagreements were resolved by discussion and, if consensus could not be reached, a third reviewer (NQ) was consulted. The relevant information reported in the articles was extracted in a standardised form developed in Excel®. This information included the study ID number, first author, year of publication, study period and location, study design, population, pollutants and pollutant increase, time-lags, units of measurement, outcomes, age of participants (if available), and association measures. The same form was used for further calculations on the original association measures, in order to obtain standardised values. The associations in the articles were reported as RR, OR, ER% and regression coefficients. In one article the authors reported the HR, but this value was an approximation derived from the OR obtained by modelling. In all cases, when we calculated a summary measure, we used the RR as the common measure of effect. If the OR was the original statistic reported in the studies, we took into account the "rare disease assumption" [14], and considered this value to be equal to the RR. If the original study reported regression coefficients, we obtained RRs using the following equation [15]:

$$RR={e}^{Coefficient}$$

The coefficient represents a unit increase in the pollutant concentration. The 95% confidence intervals around the central RR value were calculated using the standard errors and the normal approximation.

ER% values were also transformed into RRs, using the following equation [15]:

$$RR=\frac{ER\%}{100}+1$$

To obtain summary measures, the RRs associated with a given increase in pollutant in original articles (original) were first transformed to reflect the RR associated with in that level (standardised), assuming a linear relationship between concentration and risk [16], and according to the following equation:

$${RR}_{standardised}={e}^{\left(\frac{Ln({RR}_{original})\times 10}{{Increment}_{original}}\right)}$$

### Risk of bias

To assess the risk of bias in individual studies, we used a domain-based assessment tool specifically designed for air pollution studies. This tool was developed by experts convened by the World Health Organization in the context of updating the Air Quality Guidelines [2]. The items (sub-domains) in this tool are grouped into six domains: confounding, selection bias, exposure assessment, outcome measurement, missing data, and selective reporting [17]. For this review, we applied the first five domains, omitting the analysis of selective reporting. This domain is meant to assess whether the results reported in an article are different from the results to be measured, i.e. whether the results are selected. For this goal, it is recommended to identify a protocol for the study, and to compare it with the information reported in the article. Sometimes, the original research plan is given in the methods section. However, we could not find published protocols for the articles included in this review. In addition, the methods sections were not informative in this regard, leading to potential subjectivity in the judgements. Therefore, this domain was excluded from the analysis. The rationale for the analysis of each domain is not described here, but a detailed explanation for each article is provided in the Supplementary Information. Briefly, each sub-domain is classified as having a low, moderate or high risk of bias, and the worst result for a sub-domain determines the result for the whole domain. For example, if a sub-domain is rated as having a high risk of bias, the corresponding domain will be rated as having a high risk of bias, regardless of the results of the other sub-domains. For a more detailed description of the procedure, we refer to the study by Orellano and colleagues [18].

### Statistical analysis

The aim of this study was to provide a global estimate of the association between pollutant exposures and outcomes, based on studies conducted in different locations, time periods, and research designs. For this purpose, the more appropriate approach is to use random-effects models, which are able to deal with these differences and take into account the true heterogeneity. As recommended in the specialized literature, the decision to use the random-effects model over the fixed-effects model was independent of any test to measure heterogeneity. For a discussion of the use of random-effects models in this context, see the book by Hedges and colleagues, Chapter 13 [19]. When data from three or more studies could be combined, a pooled association was reported. Otherwise, the results were reported as separate association measures. To account for the difference between short-term and long-term exposure studies, we used the definitions in the WHO Air Quality Guidelines [2], where short-term exposure is calculated for a time lag between exposure and symptom onset in the order of hours to days, whereas long-term exposure is considered in the order of months to years. In some cases, pooled RRs were calculated for more than one study design when the methodologies were comparable. This was the case for cohort, panel and case-crossover studies, all of which were analysed as time-series studies. However, cross-sectional studies were analysed separately.

Heterogeneity between studies was assessed, where possible, using the prediction intervals (PI) [20]. The rule of thumb was that if the PIs included the null effect, heterogeneity between studies could be suspected [21], i.e. there will be settings where conclusions based on the CIs will not hold. We also calculated I-Squared (I2) statistics to quantify heterogeneity, using a cut-off value of 75% to detect substantial heterogeneity [22]. Caution should be taken when interpreting these statistics, as the estimate of the between-study variance can be misleading when including a small number of studies [19].

A series of subgroup and sensitivity analyses were performed to understand the influence of external variables and methodological assumptions on the summary measures. The subgroup analysis was based on study location (continent) and outcome, while the sensitivity analysis focused on the risk of bias analysis, i.e. excluding studies with a high or moderate risk of bias in a given domain. Other planned subgroup and sensitivity analyses included in the protocol could not be performed due to the small number of eligible studies considered, i.e. the sensitivity analysis by lag pattern and by study design. In addition, a leave-one-out analysis was performed to assess the influence of individual studies on the pooled relative risks.

## Results

### Description of studies

**Fig. 1:**
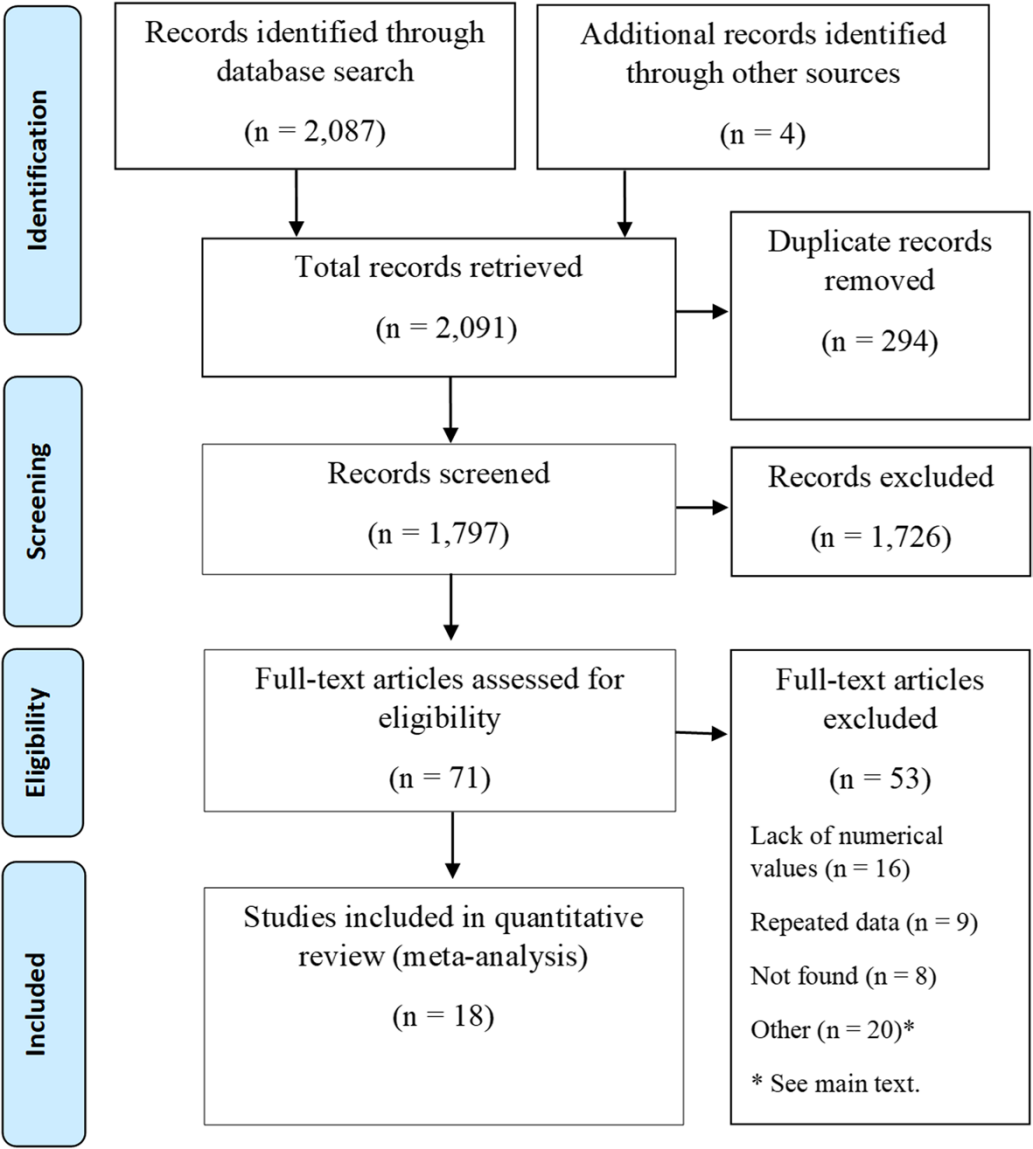
Flowchart of studies selected for inclusion in the meta-analysis

The search included 2,087 records, 1,479 in Scopus, 586 in PubMed, and 22 in regional databases, while 4 articles found in the references of reviews and other sources were added later. After duplicate exclusion, the remaining 1,797 records were screened and 71 articles were selected for full-text eligibility assessment. In the final stage, 18 articles were included for quantitative analysis. These articles included studies on the four pollutants, four outcomes, and five study designs. The reasons for excluding articles were lack of association values in the reports (16), repetition of articles or data (9), articles not available in full text (8), different exposures (6) or outcomes (2) and different modelling strategies (5), while some studies were reviews, guidelines, or predictive studies (7). The flow of the review process is shown in Fig. 1, while the studies retrieved in the search, excluded and included in the quantitative analysis are shown in Additional file 2.

A general description of the studies can be found in Table 1 and Additional file 3. The articles were published between 1987 and 2022, while the study period covered years from 1976 to 2019. The longest study period was 7 and 8 years, in two time-series studies from Norway and Brazil, while six studies lasted one year or less. Three continents, Europe, Asia and the Americas, were equally represented, although there was some variation within continents, e.g. five studies were conducted in the United States, but only one study was from Latin America. The populations studied included the general population (adults), schoolchildren, and workers. The age of the adults ranged from 18 to 65 years or from 18 to 81 years, depending on the article. Studies from Asia focused only on schoolchildren. National health surveys in the United States and Sweden were used to examine associations in the general population. Schoolchildren and groups of workers were analysed for school-loss days and work-loss days respectively. In one study [23] the workers belonged to a specific occupational group, i.e. traffic controllers. In the majority of studies, the outcomes were related to health conditions, i.e. unspecified or respiratory diseases. The exception was three articles, where the outcome was school-loss days, independent of a specific illness [24–26]. In one article, absence from work was related to mental health [27]. The total number of participants for all the articles included in this review was more than 130,000 (Additional file 3). All studies can be considered to have analysed short-term exposures, i.e. with a time lag between exposure and onset of symptoms ranging from hours to one month. Two cross-sectional studies were an exception. In one of these studies, the difference between exposure and outcome was 3 months [28]. This study is therefore in a grey area between short-term and long-term exposures. In the other study [23] it is not clear what the lag was, but it would be close to one year. In this sense, none of the studies included in this review can provide evidence of long-term associations.

**Table 1:** Description of the included studies

Study	Study period	Country	Continent	Population	Health condition
Rodrigues-Silva, 2012 [23]	2000–2007	Brazil	Americas	Workers (traffic controllers)	Illnesses
Gilliland, 2001 [29]	1996	USA	Americas	Schoolchildren	Illnesses
Hales, 2016 [24]	2011–2014	USA	Americas	Schoolchildren	All Causes
Ostro, 1987 [11]	1976-1981	USA	Americas	General	Illnesses
Ostro, 1989 [30]	1976—1981	USA	Americas	Workers	Illnesses
Rondeau, 2005 [31]	1996	USA	Americas	Schoolchildren	Illnesses
Chen, 2021 [32]	2021	China	Asia	Schoolchildren	Illnesses
Wu, 2022 [33]	2016–2017	China	Asia	Schoolchildren	Illnesses
Yang, 2019 [34]	2015–2017	China	Asia	Schoolchildren	Respiratory illnesses
Zhang, 2018 [35]	2014	China	Asia	Schoolchildren	Respiratory illnesses
Watanabe, 2021 [36]	2016–2018	Japan	Asia	Schoolchildren	Illnesses
Park, 2002 [37]	1996–1999	Korea	Asia	Schoolchildren	Illnesses
Bruyneel, 2022 [27]	2019	Belgium	Europe	Workers	Mental health
Samoli, 2017 [26]	2013–2014	Greece	Europe	Schoolchildren	All Causes
Marcon, 2014 [25]	2007–2010	Italy	Europe	Schoolchildren	All Causes
Hansen, 2000 [38]	1990–1996	Norway	Europe	Workers	Illnesses
Samakovlis, 2005 [39]	1999	Sweden	Europe	General	Respiratory illnesses
Willers, 2013 [28]	2007	Sweden	Europe	General	Respiratory illnesses

To estimate the main exposure variable, outdoor air pollution levels were measured at ground monitoring stations, which in some studies were part of established monitoring networks. In a number of studies, ground data were supplemented with information from dispersion models or satellite remote sensing data. Indoor concentrations of outdoor pollutants were not measured in the studies. Particulate matter and NO_2_ were measured as 24-h mean concentrations. For O_3_, 24-h or 8-h mean concentrations were used in longitudinal studies and 1-h maximum concentrations in cross-sectional studies.

A typical example of an ecological time-series study is that of Marcon and colleagues [25] for PM_10_ and school loss days. In this study, carried out in an Italian village, daily data on school absenteeism were obtained from registries and related to the daily average of PM_10_ concentrations using a Generalized Additive Model, with the number of absences as the dependent variable, and PM_10_ concentrations as the independent variable. A number of potential confounders were considered and included in the model, such as day of the week, influenza outbreaks, or average daily temperature. On the side of cross-sectional studies, the study by Ostro [30] can be used as a model to understand the methodology. This study makes use of the Health Interview Survey of the United States, a large cross-sectional survey covering 50,000 households that measures acute morbidity in terms of restricted activity days. The study used this database and related the outcome to data on ambient fine particulate matter and ozone, using a 2-week average of the daily readings of ozone and FP levels as independent variables in the models. In this study, potential confounders were more related to the nature of cross-sectional studies, e.g. age, sex, race, education, family income, marital status, or the presence of a chronic disease.

### Risk of bias analysis

**Fig. 2:**
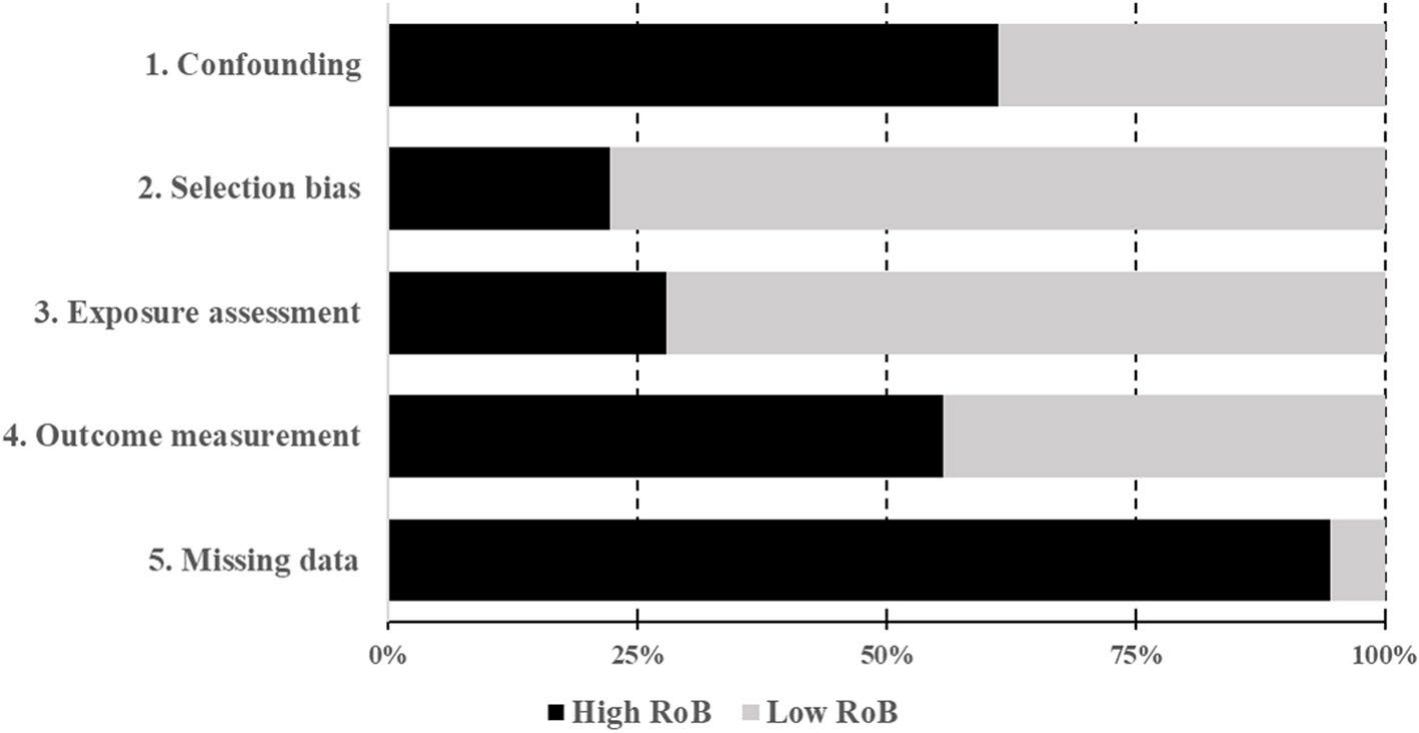
Percentage of articles with high/low risk of bias in each domain

Figure 2 shows the proportion of studies at high and low risk of bias in the selected domains. The domain with the lowest proportion of articles at high risk of bias is selection bias, with four studies judged to be at high risk of bias due to differences in the level of exposure between subjects in cross-sectional studies. The other domains all showed higher proportions of high risk of bias judgments, with the missing data domain being the topmost contributor (94%), followed by the confounding domain (61%), the outcome measurement domain (56%) and the exposure assessment domain (28%). For the confounding domain, the main concerns were related to the lack of adjustment for control variables, e.g. seasonality or weather variables in time-series studies, and also with uncertainties in the measurement of these confounders. Outcome measurement issues were related to the use of self-reported outcome data in surveys. The risk of bias related to the missing data domain was judged as high in all but one study, where missing or excluded data were reported as occasional. All other articles did not report missing exposures, outcomes or both. Overall, the included articles had at least one reason for concern about the risk of bias, and 13 articles had a high risk of bias in more than one domain. The risk of bias assessments and the rationale for each study can be found in Additional file 4, on a case-by-case basis.

### Meta-analysis

Given the diversity of exposures, outcomes and study designs, each exposure-outcome pair was pooled using a maximum of seven articles. Because of the small number of studies, some of the subgroup and sensitivity analyses were not performed, depending on the pollutant or the outcome measure. When pooled effects were calculated, RRs for a 10 µg/m^3^ increase were reported for each pollutant and study design. The association measures, as reported in the original articles, are shown in Additional file 3, while the pooled effects are shown in Table 2.

**Table 2:** Pooled relative risks

Pollutant	Outcome	Study type	Articles	N	RR (95%CI) (Random-effects model)	80%PI	I2
PM_10_	WLD and SLD combined	TS or CHS	7	7	1.0175 (1.0040–1.0311)	0.9964–1.0389	69%
PM_10_	SLD	TS or CHS	6	6	1.0149 (1.0017–1.0283)	0.9943–1.0360	69%
PM_2.5_	SLD	TS, PS or CCO	6	8	1.0173 (1.0056–1.0290)	0.9951–1.0399	99%
PM_2.5_	RAD	CS	1	6	1.0493 (1.0288–1.0702)	1.0092–1.0910	91%
PM_2.5_	RRAD	CS	1	6	1.1575 (1.1207–1.1956)	1.1210–1.1952	10%
PM_2.5_	MRAD	CS	1	6	1.0710 (0.8862–1.2943)	0.7243–1.5835	99%
PM_2.5_	WLD	CS	1	6	1.0699 (0.9971–1.1481)	0.9276–1.2341	97%
NO_2_	WLD and SLD combined	TS, CHS or CCO	7	7	1.0075 (0.9915–1.0237)	0.9800–1.0358	77%
NO_2_	SLD	TS, CHS or CCO	6	6	1.0085 (0.9909–1.0264)	0.9782–1.0398	80%
O_3_	SLD	TS, PS or CHS	4	4	1.0134 (0.9715–1.0571)	0.9381–1.0948	87%
O_3_	RRAD	CS	1	6	0.9595 (0.9188–1.0020)	0.8838–1.0416	86%
O_3_	MRAD	CS	1	6	1.0150 (0.9762–1.0552)	0.9377–1.0986	97%

*PM*_*10*_ particulate matter with aerodynamic diameters less or equal than 10 μm, *PM*_*2.5*_ particulate matter with aerodynamic diameters less or equal than 2.5 μm, *NO*_*2*_ nitrogen dioxide, *O*_*3*_ ozone, *WLD* work-loss day, *SLD* school-loss day, *RAD* restricted activity day, *RRAD* respiratory-related restricted activity day, *MRAD* minor restricted activity day, *TS* time-series study, *CHS* cohort study, *PS* panel study, *CCO* case-crossover study, *CS* cross-sectional study, *Articles* number of articles included, *N* number of effect sizes, *RR* pooled relative risk, calculated for a 10 μg/m^3^ increase in the pollutant level, *95% CI* 95% confidence interval, *80% PI* 80% prediction interval, *I2* I-Squared values

### Particulate matter (PM_10_)

**Fig. 3:**
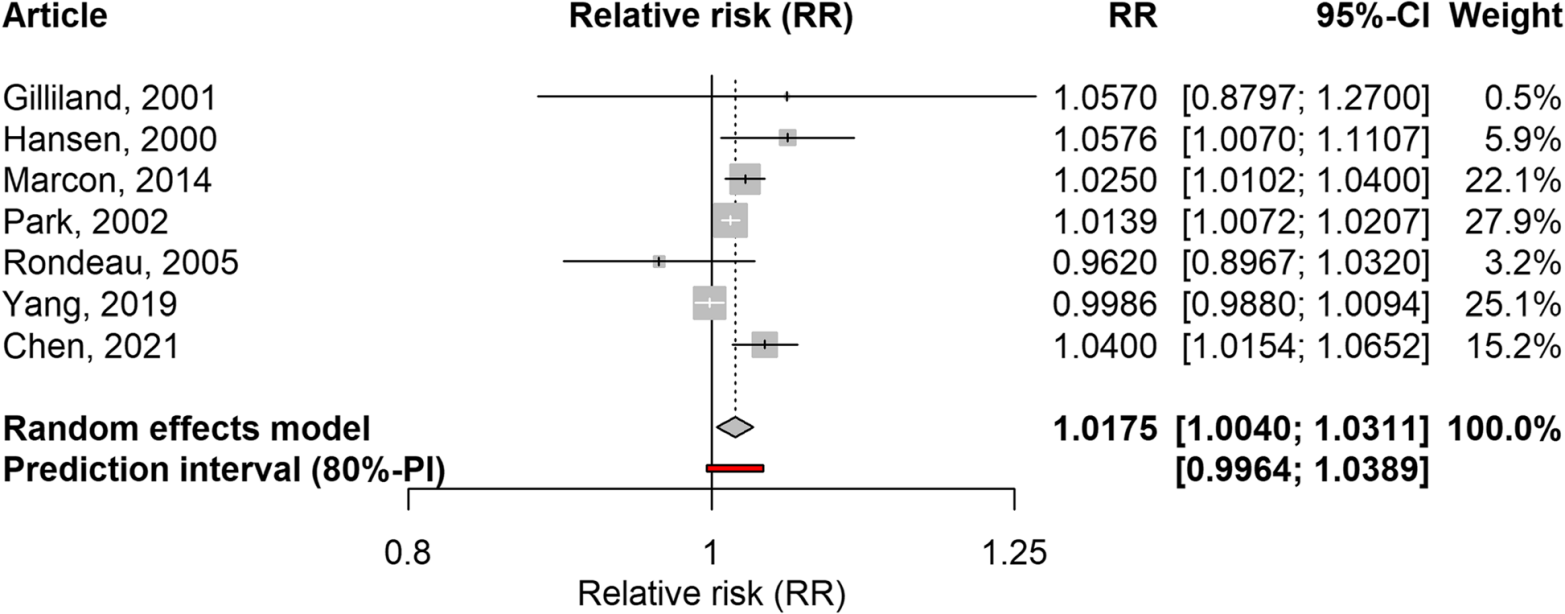
Forest plot of 6 time-series studies and one cohort study assessing the association between PM_10_ and restricted activity days (work-loss days and school-loss days combined). Relative risks for a 10 µg/m^3^ increase in PM_10_ level

**Fig. 4:**
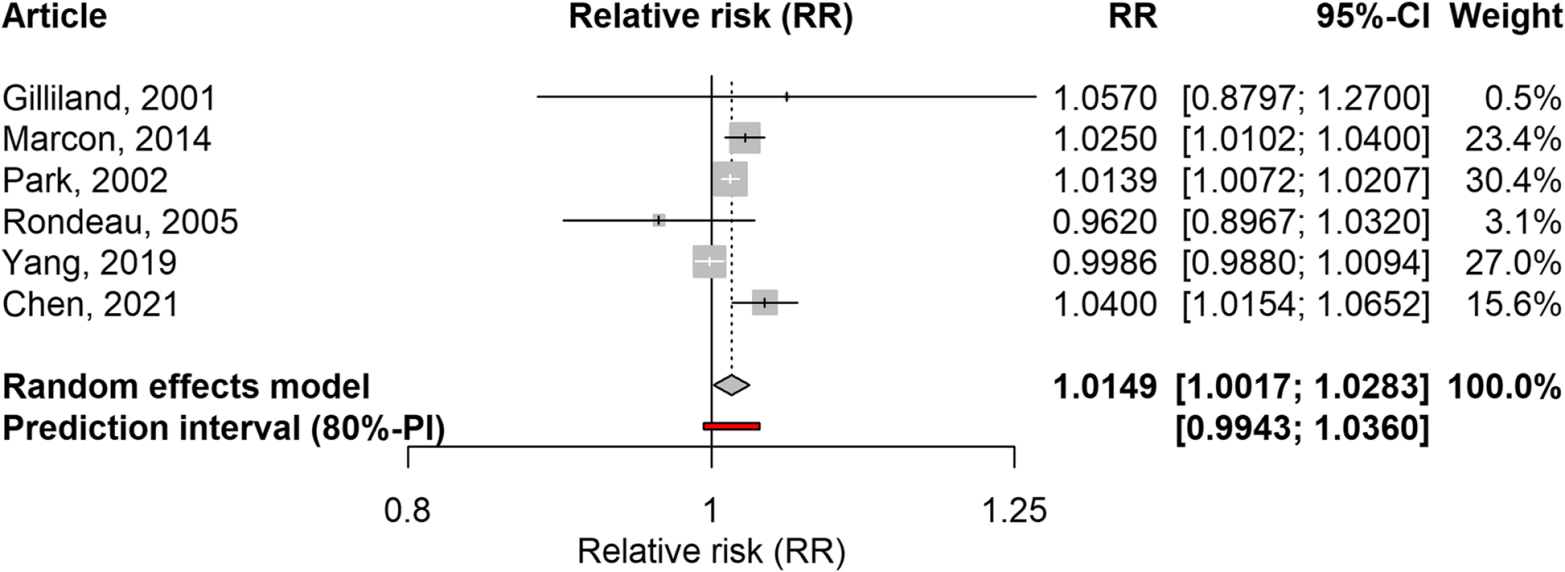
Forest plot of 5 time-series studies and one cohort study assessing the association between PM_10_ and school-loss days. Relative risks for a 10 µg/m^3^ increase in PM_10_ level

Six ecological time-series studies and one cohort study examined the association between PM_10_ and school-loss days or work-loss days in children and adults respectively [25, 29, 31, 32, 34, 37, 38]. The time lags ranged from zero (same day) to five days, with no predominance of one time lag over the others. The association was significant when considering both outcomes together (RR: 1.0175; 95%CI: 1.0040–1.0311; 80%PI: 0.9964–1.0389; I2: 69%), or when considering only school-loss days (RR: 1.0149; 95%CI: 1.0017–1.0283; 80%PI: 0.9943–1.0360; I2:69%), but not significant when considering only studies conducted in Asia. Individual and pooled values are shown in the forest plots of Figs. 3 and 4. The only ecological time-series study focusing on work-loss days [38] found a significant association. The sensitivity analysis showed that when only studies with a low risk of bias in the outcome domain were included, the RR value did not change the direction of the RRs (Table 3). In the main analysis, the PIs included the null effect, indicating that there was some degree of heterogeneity between studies, while the I2 was above the cut-off value for detecting relevant heterogeneity. The leave-one-out analysis showed that excluding any of the articles gave similar results regarding the direction of the RRs (Supplementary Table 2, Additional file 1).

**Table 3:** Sensitivity analysis. Pooled relative risks considering low RoB time-series studies

Pollutant	Outcome	Domain	Articles	N	RR (95%CI) (Random-effects model)
PM_10_	WLD and SLD combined	Confounding	4	4	1.0120 (0.9997–1.0244)
PM_10_	WLD and SLD combined	Outcome	4	4	1.0240 (0.9923–1.0567)
PM_2.5_	SLD	Confounding	3	5	1.0146 (0.9987–1.0307)
PM_2.5_	SLD	Outcome	2	4	1.0124 (0.9985–1.0265)
NO_2_	WLD and SLD combined	Confounding	3	3	1.0065 (0.9997–1.0133)
NO_2_	WLD and SLD combined	Outcome	3	3	0.9796 (0.9631–0.9963)

*PM*_*10*_ particulate matter with aerodynamic diameters less or equal than 10 μm, *PM*_*2.5*_ particulate matter with aerodynamic diameters less or equal than 2.5 μm, *NO*_*2*_ nitrogen dioxide, *WLD* work-loss day, *SLD* school-loss day, *Articles* number of articles included, *N* number of effect sizes, *RR* pooled relative risk, calculated for a 10 μg/m^3^ increase in the pollutant level, *95% CI* 95% confidence interval

As for other study designs, a cross-sectional study [28] evaluating restricted activity days in relation to locally generated wear particles reported significant values, as did a study analysing work-loss days in traffic-controllers [23].

### Particulate matter (PM_2.5_)

**Fig. 5:**
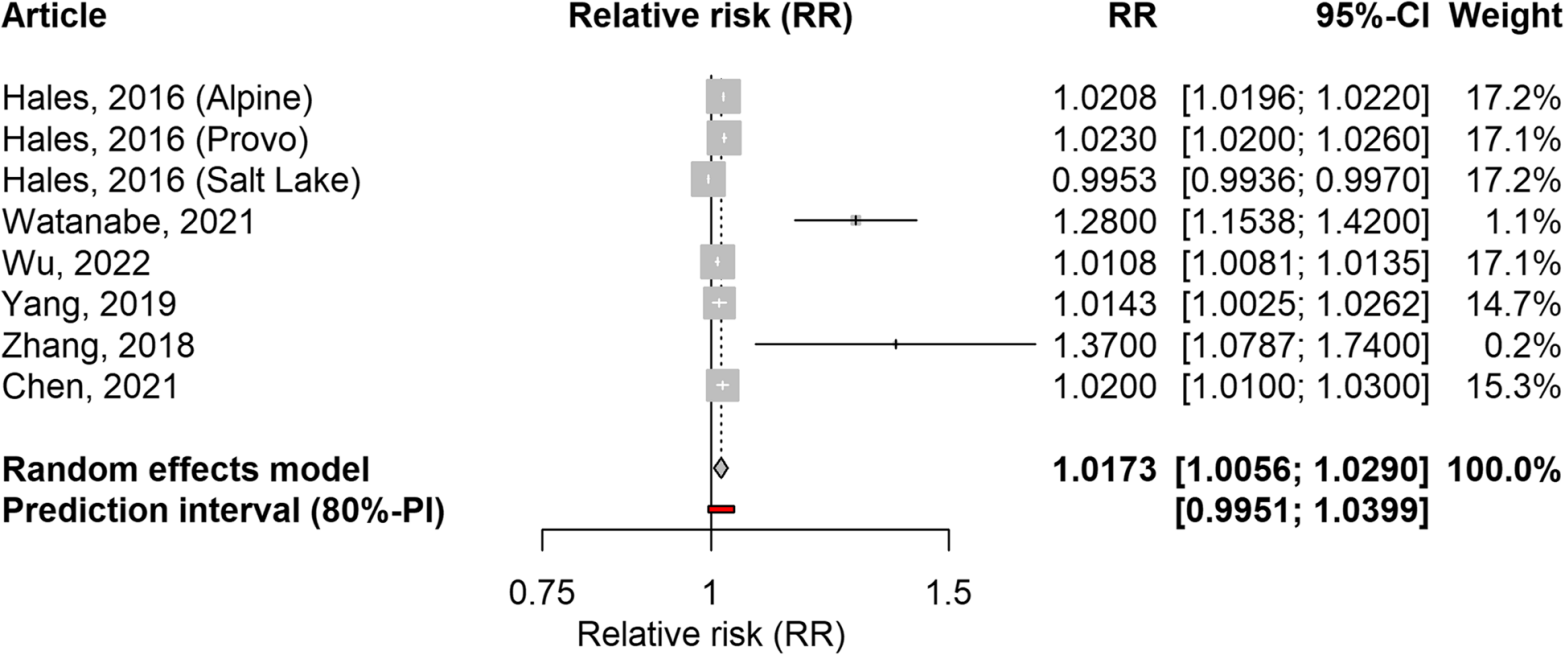
Forest plot of four time-series studies, one case-crossover study, and one panel study (8 effect sizes) evaluating the association between PM_2.5_ and school-loss days. Relative risks for a 10 µg/m^3^ increase in PM_2.5_ level

Based on data from four ecological time-series studies, one case-crossover study, and one panel study [24, 32–36], the association between PM_2.5_ and school-loss days was significant (RR: 1.0173; 95%CI: 1.0056–1.0290; 80%PI: 0.9951–1.0399; I2: 99%). The majority of studies considered exposure and outcome on the same day or with a lag of one day. The forest plot is shown in Fig. 5. Heterogeneity was observed among the included studies. The association remained significant for studies from Asia (RR: 1.0220; 95%CI: 1.0052–1.0390; 80%PI: 0.9947–1.0500; I2: 86%), but not for the study conducted in three cities in North America. In the sensitivity analysis, the RR decreased slightly when studies with a high risk of bias in the outcome or confounding domains were excluded (Table 3). The leave-one-out analysis showed that excluding any of the included studies did not affect the results (Supplementary Table 3, Additional file 1).

A single case-crossover study examined the association with work-loss days and reported significant results, although this study was focused on absenteeism related to mental health problems [27]. The associations between PM_2.5_ and restricted activity days, minor restricted activity days, and work-loss days, were analysed in two cross-sectional studies [11, 30], which reported these associations for the period 1976–1981 in the United States. These results can, under certain conditions, be considered to be related to short-term exposures, because lags of two and four weeks between exposure and outcome were used. These studies reported 6 relative risk values, one for each year. We performed a meta-analysis across the years reported in these articles to calculate summary estimates, and found significant values for restricted activity days (RR: 1.0493; 95%CI: 1.0288–1.0702; 80%PI: 1.0092–1.0910; I2: 91%) and for respiratory-related restricted activity days (RR: 1.1575; 95%CI: 1.1207–1.1956; 80%PI: 1.1210–1.1952; I2: 10%), but not for minor restricted activity days or work-loss days. In the significant associations, the heterogeneity was not relevant, although a high I2 value was estimated for restricted activity days. As these estimates were obtained from two studies analysing the same population over the years, subgroup and sensitivity analyses were not performed. Individual and pooled values are shown in Supplementary Figs. 1 to 4, Additional file 1.

### Nitrogen dioxide (NO_2_)

**Fig. 6:**
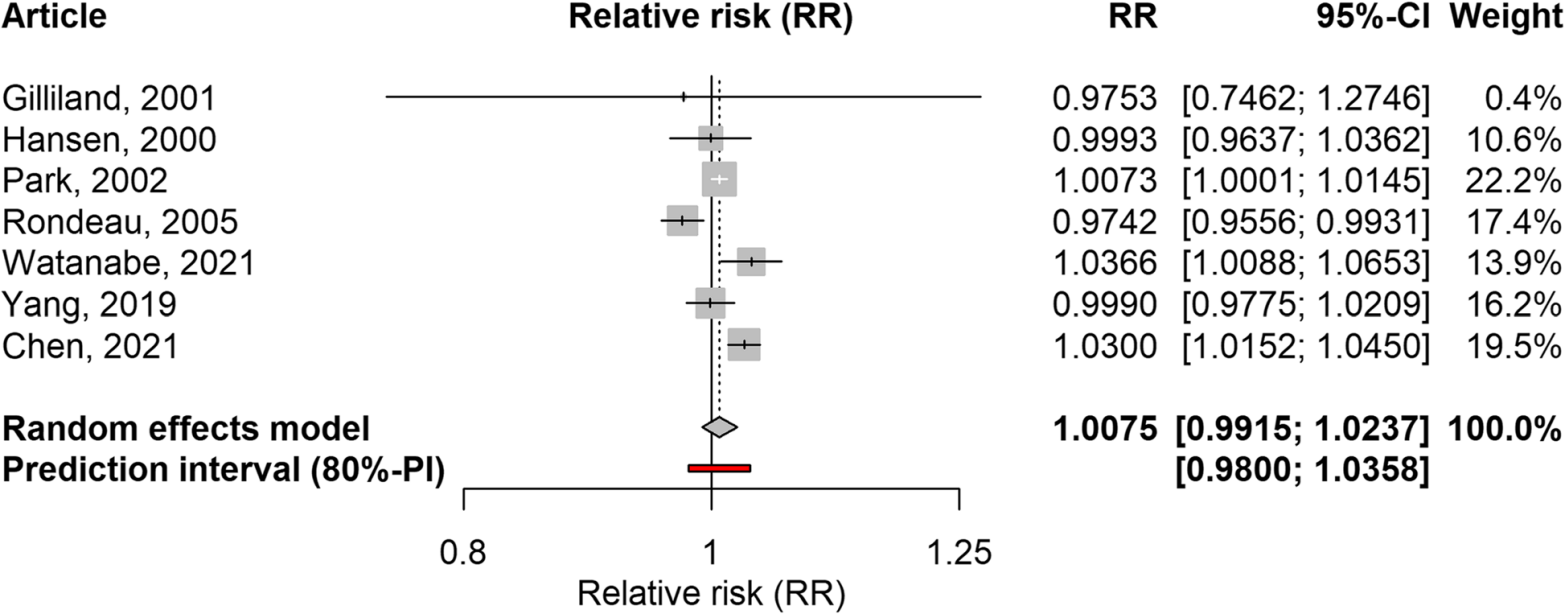
Forest plot of 6 time-series studies and one cohort study evaluating the association between NO_2_ and restricted activity days (work-loss days and school-loss days combined). Relative risks for a 10 µg/m^3^ increase in NO_2_ level

**Fig. 7:**
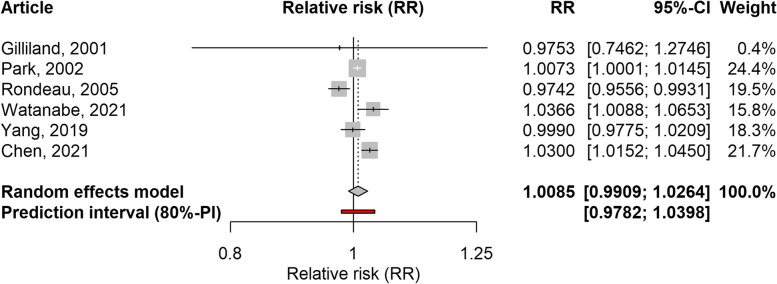
Forest plot of 5 time-series studies and one cohort study evaluating the association between NO_2_ and school-loss days. Relative risks for a 10 µg/m^3^ increase in NO_2_ level

Seven articles analysing the effect of NO_2_ were included, five ecological time-series, one cohort study, and one case-crossover study design [29, 31, 32, 34, 36–38]. As with PM_2.5_, most studies considered exposure and outcome on the same day or with a lag of one day. The association between NO_2_ and school-loss and work-loss days was not significant when considering both outcomes together (RR: 1.0075; 95%CI: 0.9915–1.0237; 80%PI: 0.9800–1.0358; I2: 77%), or when considering only school-loss days (RR: 1.0085; 95%CI: 0.9909–1.0264; 80%PI: 0.9782–1.0398; I2: 80%). The forest plot for these analyses is shown in Figs. 6 and 7. On the other hand, the association was significant when only studies conducted in Asia were included (RR: 1.0168; 95%CI: 1.0010–1.0327; 80%PI: 0.9876–1.0467; I2: 74%). When studies with a high risk of confounding or outcome bias were excluded, the RR value was markedly reduced or even changed its direction compared to the original value (Table 3). Based on the information provided by the PIs and I2 values, some heterogeneity between studies was also found for this pollutant. The previously mentioned study focusing on absenteeism related to mental health [27] reported a significant association with NO_2_. The leave-one-out analysis again showed that excluding any of the included studies did not affect the results (Supplementary Table 4, Additional file 1). The exception was the exclusion of one cohort study carried out in the United States, which reported a surprising inverse association with school loss days [31].

A study from Sweden [39] used restricted activity days related to respiratory diseases as the outcome measure and found significant associations using a Poisson model. This study was based on a national survey. On the other hand, the study analysing work-loss days among traffic controllers [23] found no significant association for NO_2_.

### Ozone (O_3_)

**Fig. 8:**
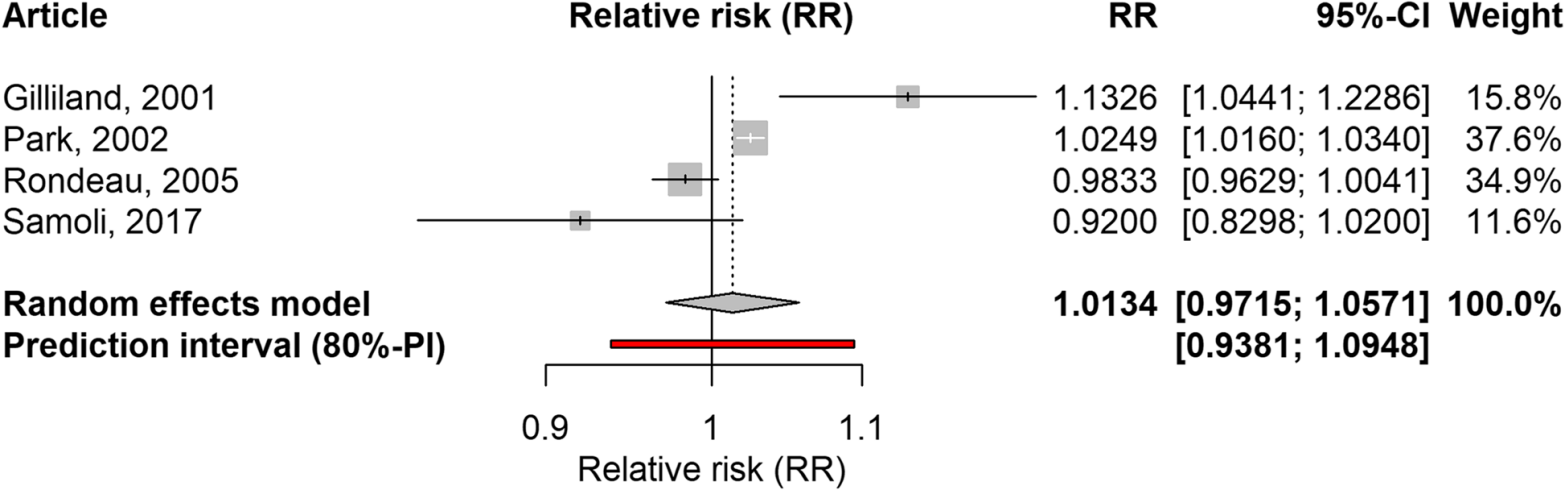
Forest plot of 2 time-series studies, one panel and one cohort study evaluating the association between O_3_ and school-loss days. Relative risks for a 10 µg/m^3^ increase in O_3_ level

Two ecological time-series studies, one cohort study and one panel study evaluated the effect of O_3_ on school-loss days [26, 29, 31, 37]. The time lags ranged from zero (same day) to five days. The association was positive but not significant (RR: 1.0134; 95%CI: 0.9715–1.0571; 80%PI: 0.9381–1.0948; I2: 87%) (Fig. 8). Similarly, the case-crossover study assessing work-loss days related to mental health [27] did not find a significant association. Leave-one-out analysis failed to identify an influential study whose exclusion might change these results (Supplementary Table 5, Additional file 1).

In the same cross-sectional study evaluating the effect of PM_2.5_ exposure in the United States, O_3_ was also analysed [30], resulting in non-significant summary associations for respiratory-related restricted activity days and for minor restricted activity days. The forest plots are shown in Supplementary Figs. 5 and 6, Additional File 1. In the same line, the study evaluating work-loss days among traffic controllers [23] did not find a significant association with O_3_.

## Discussion

### Summary of main findings

Our systematic review identified 18 articles that analysed the association between air pollution levels and different outcomes related to restricted activity days. These studies followed ecological time-series, case-crossover, panel, cohort and cross-sectional study designs, were conducted in different locations and regions, and covered a period of 43 years from 1976 to 2019. We found that short-term associations were significant in time-series studies for PM_10_ when school-loss days and work-loss days were considered together as outcomes, and for PM_2.5_ when school-loss days were considered, but not for NO_2_ or O_3_. These associations were found to be heterogeneous across studies, which introduces some uncertainty in the pooled relative risks and reflects the expected variability of this relationship. This heterogeneity would be partly related to differences in studies conducted in different continents, but other sources of heterogeneity could not be investigated. However, given the small number of studies included in the statistical analysis to assess heterogeneity, these statistical values may not represent a biologically relevant heterogeneity. On the other hand, the results of this study were robust to sensitivity analyses based on the risk of bias in different domains. As for short-term associations in cross-sectional studies, these were significant for PM_2.5_ and restricted activity days and for PM_2.5_ and respiratory-related restricted activity days. The associations with O_3_ were not significant and, surprisingly, in some years the increase in this pollutant resulted in protective effects. This paradoxical result may be related to measurement error in averaging the time of exposure to the pollutant, among other factors. Long-term associations could not be examined because only two studies evaluated the associations between exposures and outcomes in the long term, in the order of months.

### Historical overview of studies on restricted activity days

The association between restricted activity days and various individual and socioeconomic predictors was first investigated in the late 1960s with a series of reports derived from household interviews in the US National Health Interview Survey. For example, a 1972 report [9] found an estimate of 3 billion days of restricted activity in the general population (all ages), or an average of 15.3 days per person/year, for the year 1968. These associations were modulated by the effect of age, sex, place of residence, income, and employment status. According to this report, more limited activity is to be expected for the female population, for people living in metropolitan areas, for families with lower income levels and for the non-working population (e.g. retired people). In 1996, the same survey showed similar values for restricted activity days in the general population (14.5 days per person/year), but the survey report also took into account restricted activities by type of outcome, i.e. 5.9 days for bed disability, 4.8 days for work-loss days for employed persons and 4 school-loss days for children and adolescents aged 5–17 years of age [40]. This last report was by then more detailed in terms of acute and chronic conditions leading to restricted activity days, e.g. asthma, infectious diseases, injuries, to name but a few.

It was not until the 1980s that the US National Health Interview Survey database was explored to associate restricted activity days with total suspended particulate matter (TSP) and sulphur dioxide, and association measures were obtained using statistical regression models [41]. In a series of papers published between 1983 and 1989, Ostro and colleagues reported an association between particulate matter, sulphur dioxide, ozone and several outcomes, including restricted activity days, minor restricted activity days, and work-loss days. However, earlier work had examined the effect of air pollution on outcomes that fall under the umbrella of restricted activity days. For example, an article from 1967 analysed the annual reports from the Ministry of Pensions and National Insurance in the United Kingdom, and found a significant correlation between incapacity to work due to bronchitis and local air pollution [42]. Another article, using data from the United States, correlated children's school absenteeism with the exposure to various pollutants [43]. In general, however, studies prior to the 1980s do not report association values between exposure levels and outcomes, as in the above-mentioned examples. In more recent years, a number of studies with different epidemiological designs, e.g. cross-sectional, case-crossover, ecological time-series, have been developed to assess the association between air pollution and restricted activity days, work-loss days and school-loss days in several countries in Europe, Asia and North-America. The procedures for the analysis included rigorous regression techniques that allowed the access to more detailed results. In parallel with epidemiological studies looking at empirical associations between air pollution and restricted activity days, a number of studies and reports have been developed to make predictions about the public-health impact of traffic-related air pollution, with outcomes including mortality, hospital admissions, and restricted activity days. For example, a modelling study by Kunzli and colleagues [44] used relative risks calculated from an observational study [45] to estimate the impact of PM_10_. Another modelling study used a Health Impact Assessment approach to estimate the potential benefits of reducing ozone levels in California [46]; this study used the empirical effect sizes reported by Ostro and Rothschild [30] for the prediction models. More recently, two modelling studies [47, 48] have simulated the health benefits of reducing air pollution emissions, both studies using the empirical effect sizes reported by Ostro [11]. Given the extraordinary importance of large-scale health impact assessments and the strong reliance on reliable relative risk estimates, more observational studies and meta-analyses are needed to produce robust evidence. It is becoming increasingly clear that the availability of reliable data is crucial to obtaining valid estimates of disease burden for environmental diseases [49]. In this systematic review, we found little evidence of long-term associations between pollutants and days of restricted activity, although this is an important aspect of the relationship between these variables. Long-term exposures may not be the simple sum of a number of short-term effects, and this influence should be analysed differently. On the other hand, the number of short-term studies included in this review was not sufficient to perform more specific analyses, i.e. subgroup analyses that take into account variability related to sex, age, type of outcome and other important factors.

### Restricted activity days in context

A growing body of scientific literature supports the associations between air pollution and restricted activity days by validating the associations with more objective measures, i.e. morbidity and mortality. In this review, a number of studies focused on respiratory-related restricted activity days, as these conditions are at the top of the list of causes of absence from school or work. In this sense, the published evidence of associations between air pollution and respiratory diseases provides more insight into this relationship. A recent study in China [50] showed an association between PM_10_ and PM_2.5_ and the common cold in young adults without a history of asthma or allergic rhinitis, with RRs of 1.17 and 1.28, respectively, for an increase of 10 μg/m^3^. More serious outcomes have also been associated with exposure to outdoor air pollution: a systematic review and meta-analysis [51] found a 0.4% and 1% increase in the number of hospitalisations for pneumonia associated with a 10 μg/m^3^ increase in PM_10_ and PM_2.5_, respectively. With regard to chronic diseases, asthma and COPD have been identified in the past as causes of absenteeism from school and work, and there is a large body of literature on the subject. Systematic reviews and meta-analyses have collected and analysed evidence of associations between air pollution and asthma and COPD exacerbations [52–54]. In terms of more serious outcomes, short- and long-term exposures to PM_10_, PM_2.5_ and other pollutants have recently been associated with respiratory mortality and mortality from other causes [18, 55].

### Risk of bias assessment

All studies showed problems in at least one domain, i.e. a high risk of bias, according to our criteria. Moreover, the majority of studies showed a high risk of bias in at least two domains, with the missing data domain being the most common. In this sense, it is not uncommon for observational studies not to report the number of missing observations or to lack procedures for imputing missing data. Furthermore, if the missing observations were randomly distributed, the effect on associations would not be highly relevant. More importantly, two other domains showed a high risk of bias in more than 50% of the articles: outcome measurement and confounding. The former is mainly related to the participants' self-reporting of the outcome, i.e. we considered that the risk of bias might have been a concern in those studies where school or work absence was self-reported. This is in contrast to administrative reports from companies, schools or employment registers, where the information is more trustworthy. Similarly, a high risk of bias in the confounding domain means that the association may be due to observed or unobserved confounders rather than to exposure to air pollution. For example, the short-term influence of ambient temperature on human morbidity is well known [56], and at the same time the association between temperature and air pollutant concentrations has been well-established in several studies; see, for example, Analitis and colleagues (2018) [57]. This double association with the exposure and the outcome makes temperature a perfect candidate for confounding. Other potential confounders are the day of the week or holidays, especially in short-term time-series studies. In addition, the inclusion of a smooth function of time can adjust for seasonal and long-term trends, which in turn, is a form of adjustment for unmeasured confounders that vary smoothly over time [15]. All of these potential confounders had to be included in the regression models for an ecological time-series study to be considered low risk of bias. For case-crossover studies, time-dependent variables were considered to be controlled for by design [58]. Finally, for cross-sectional, panel and cohort studies, the confounders to be included were age and sex. Beyond these potential biases, which were assessed using the risk of bias tool, the importance of exposure misclassification cannot be overlooked. For our review, we considered measuring exposure to air pollution using monitoring stations as a proxy for individual exposure to polluted air. However, the use of ambient exposures rather than individual exposures could be subject to bias, i.e. individual exposures could be higher than ambient exposures, leading to an underestimation of threshold concentrations [59]. Overall, when the same estimates were calculated using only studies with low risk of bias, the results showed no change in the direction of the associations. Other sensitivity analyses based on study design or time lag could not be performed, so the uncertainty about methodological decisions and assumptions remains open.

### Publication bias

When using funnel plots or regression tests to assess the potential presence of publication bias, it is recommended to use a minimum number of 10 studies, or substantially more than 10 studies in the presence of heterogeneity [60]. In this review, it was not possible to reach this minimum number of studies for each exposure-outcome pair, and therefore bias due to unpublished or grey research could not be excluded. As we were not able to perform a test to assess publication bias, we made additional efforts to avoid this problem. In this sense, we implemented a comprehensive search strategy that included regional databases as sources of both scientific articles and grey literature. We also included studies without language restrictions to avoid language bias [61]. However, 8 studies originally selected for inclusion in this analysis could not be found, which represents a significant proportion of the studies finally included and should be seen as another limitation of this work. Six of these studies were published in the 1970s, which may explain the difficulty in finding the full texts. In the other two articles, conducted in France and Germany, groups of children exposed to air pollution in polluted areas were more prone to respiratory diseases [62] and to school absenteeism [63]. Another way to increase the sensitivity of the review process was to include different study designs, i.e. ecological time-series, case-crossover, panel, cohort, and cross-sectional studies. This inclusion had the disadvantage of requiring additional assumptions to pool the different measures of association, and in most cases the analysis was split on the basis of design type. Additional efforts were made to assess the suitability of different studies for inclusion in the same analysis, and these decisions and assumptions are always subject to error.

### Summary of limitations

Earlier in this section we discussed various limitations and caveats of the evidence provided by this systematic review and meta-analysis. In this sub-heading, we provide a brief summary of these and other possible limitations. First, although restricted activity days and its related school and work absenteeism were outcomes that were investigated at an early stage in the assessment of the health effects of air pollution, the number of studies dealing with these outcomes was small, and some studies could not be found. As mentioned above, the small number of studies prevented us from assessing publication bias, the impact of methodological assumptions on the results, and the influence of moderator variables on the heterogeneity. Second, the diversity of study designs, an advantage in terms of reducing the possibility of publication bias, led to difficulties in interpreting and pooling association values and potential pitfalls in the assumptions. Third, all articles were considered to be at high risk of bias in at least one domain, with some of these domains being at high risk in the majority of the articles. Failure to report missing data, failure to include all relevant confounders, and failure to measure the outcome were the most common reasons for considering the observed risk of bias to be high. Fourth, some individuals may have been exposed to both indoor and outdoor air pollution, but only outdoor exposure was considered in this review. In this sense, some unavoidable bias is to be expected. Finally, as with many other reviews on air pollution and health, there were differences in geographical representativeness, with Europe and North America contributing a high proportion of articles and regions with a majority of low- and middle-income countries less represented. It is worth noting that, given the important influence of environmental variables on the associations between outdoor air pollution and human health, research in different locations with different environmental conditions is crucial to fully understand these relationships on a global scale. However, air pollution is a global problem, and reference values for air pollution levels should not be addressed to countries according to their income; the urgency of the situation justifies the establishment of air quality policies, especially in developing countries [64].

## Conclusions

Our study allowed the calculation of pooled effect sizes between selected ambient air pollutants and restricted activity days, including school and work absenteeism. These associations were significant for PM_10_ and PM_2.5_, even with different study designs and outcomes. The small number of studies included in the review, and the large proportion of studies with a high risk of bias introduce some uncertainty into these associations. However, the proven associations between the exposure to ambient air pollutants and human diseases that usually lead to restricted activity days, e.g. asthma, COPD, viral respiratory infections, as reported in scientific articles and reports, is a second-hand evidence that lends plausibility to the associations of air pollutants with restricted activity days. Despite the limitations of this study, the association between air pollution and restricted activity days is worth considering when modelling and calculating the adverse effects of air pollution on human health.

## Supplementary Information

**Additional file 1: Supplementary Table 1.** Search strategy: the example of MEDLINE via PubMed. **Supplementary Table 2.** Influential analysis for PM_10_ and work-loss/school-loss days. **Supplementary Table 3.** Influential analysis for PM_2.5_ and school-loss days. **Supplementary Figure 1.** Forest plot of one cross-sectional study (6 effect sizes) evaluating the association between PM_2.5_ and restricted activity days. Relative risks for a 10 µg/m^3^ increase in PM_2.5_ level. **Supplementary Figure 2.** Forest plot of one cross-sectional study (6 effect sizes) evaluating the association between PM_2.5_ and respiratory-related restricted activity days. Relative risks for a 10 µg/m^3^ increase in PM_2.5_ level. **Supplementary Figure 3.** Forest plot of one cross-sectional study (6 effect sizes) evaluating the association between PM_2.5_ and minor restricted activity days. Relative risks for a 10 µg/m^3^ increase in PM_2.5_ level. **Supplementary Figure 3.** Forest plot of one cross-sectional study (6effect sizes) evaluating the association between PM_2.5_ and minor restricted activity days. Relative risks for a 10 µg/m^3^ increase in PM_2.5_ level. **Supplementary Figure 4.** Forest plot of one cross-sectional study (6 effect sizes) evaluating the association between PM_2.5_ and work-loss days. Relative risks for a 10 µg/m^3^ increase in PM_2.5_ level. **Supplementary Table 4.** Influential analysis for NO_2_ and work-loss/school-loss days. **Supplementary Figure 5.** Forest plot of one cross-sectional study (6 effect sizes) evaluating the association between O_3_ and respiratory-related restricted activity days. Relative risks for a 10 µg/m^3^ increase in O_3_ level. **Supplementary Figure 6.** Forest plot of one cross-sectional study (6 effect sizes) evaluating the association between O_3_ and minor restricted activity days. Relative risks for a 10 µg/m^3^ increase in O_3_ level.

**Additional file 2. **Studies included and excluded.

**Additional file 3. **Information extracted from the studies.

**Additional file 4. **Risk of bias assessment.

## Data Availability

The datasets supporting the conclusions of this article are included within the article and its additional files.

## Abbreviations

PM_10_: Particulate matter with aerodynamic diameters less or equal than 10 μm
PM_2.5_: Particulate matter with aerodynamic diameters less or equal than 2.5 μm
WLD: Work-loss day
SLD: School-loss day
RAD: Restricted activity day
RRAD: Respiratory-related restricted activity day
MRAD: Minor restricted activity day
TS: Time-series study
CCO: Case-crossover study
CS: Cross-sectional study
N: Number of effect sizes
RR: Pooled relative risk, calculated for a 10 μg/m^3^ increase in the pollutant level
95% CI: 95% Confidence interval
80% PI: 80% Prediction interval
I2: I-Square values

## References

[1] Khomenko S, Cirach M, Pereira-Barboza E, Mueller N, Barrera-Gómez J, Rojas-Rueda D (2021). Health impacts of the new WHO air quality guidelines in European cities. Lancet Planet Health.

[2] WHO global air quality guidelines: Particulate matter (PM2.5 and PM10), ozone, nitrogen dioxide, sulfur dioxide and carbon monoxide [Internet]. Geneva: World Health Organization; 2021 [cited 2022 Aug 24]. Available from: http://www.ncbi.nlm.nih.gov/books/NBK574594/. 34662007

[3] Hoffmann B, Boogaard H, de Nazelle A, Andersen ZJ, Abramson M, Brauer M (2021). WHO Air Quality Guidelines 2021-Aiming for Healthier Air for all: A Joint Statement by Medical, Public Health, Scientific Societies and Patient Representative Organisations. Int J Public Health.

[4] Morelli X, Gabet S, Rieux C, Bouscasse H, Mathy S, Slama R (2019). Which decreases in air pollution should be targeted to bring health and economic benefits and improve environmental justice?. Environ Int.

[5] Yin H, Brauer M, Zhang JJ, Cai W, Navrud S, Burnett R (2021). Population ageing and deaths attributable to ambient PM2·5 pollution: a global analysis of economic cost. Lancet Planet Health.

[6] Chen S, Bloom DE (2019). The macroeconomic burden of noncommunicable diseases associated with air pollution in China. PLoS ONE.

[7] Kinman G (2019). Sickness presenteeism at work: prevalence, costs and management. Br Med Bull.

[8] Allen CW, Diamond-Myrsten S, Rollins LK (2018). School Absenteeism in Children and Adolescents. Am Fam Physician.

[9] Wilder MH (1972). Disability days, United States-1968. Vital Health Stat.

[10] Hubbell BJ, Hallberg A, McCubbin DR, Post E (2005). Health-Related Benefits of Attaining the 8-Hr Ozone Standard. Environ Health Perspect.

[11] Ostro BD (1987). Air pollution and morbidity revisited: A specification test. J Environ Econ Manag.

[12] Morgan RL, Whaley P, Thayer KA, Schünemann HJ (2018). Identifying the PECO: A framework for formulating good questions to explore the association of environmental and other exposures with health outcomes. Environ Int.

[13] Atkinson RW, Cohen A, Mehta S, Anderson HR (2012). Systematic review and meta-analysis of epidemiological time-series studies on outdoor air pollution and health in Asia. Air Qual Atmos Health.

[14] Greenland S, Thomas DC (1982). On the need for the rare disease assumption in case-control studies. Am J Epidemiol.

[15] Peng RD, Dominici F. Statistical Methods for Environmental Epidemiology with R: A Case Study in Air Pollution and Health. New York ; London; 2008.

[16] Shah ASV, Langrish JP, Nair H, McAllister DA, Hunter AL, Donaldson K (2013). Global association of air pollution and heart failure: a systematic review and meta-analysis. Lancet.

[17] Morgan RL, Thayer KA, Santesso N, Holloway AC, Blain R, Eftim SE (2019). A risk of bias instrument for non-randomized studies of exposures: A users’ guide to its application in the context of GRADE. Environ Int.

[18] Orellano P, Reynoso J, Quaranta N, Bardach A, Ciapponi A. Short-term exposure to particulate matter (PM10 and PM2.5), nitrogen dioxide (NO2), and ozone (O3) and all-cause and cause-specific mortality: Systematic review and meta-analysis. Environ Int. 2020;142:105876. 10.1016/j.envint.2020.105876 32590284

[19] Hedges LV, Higgins JPT, Rothstein HR, Borenstein M. Introduction to Meta-Analysis. Chichester, U.K; 2009.

[20] Borenstein M (2020). Research Note: In a meta-analysis, the I2 index does not tell us how much the effect size varies across studies. J Physiother.

[21] IntHout J, Ioannidis JPA, Rovers MM, Goeman JJ (2016). Plea for routinely presenting prediction intervals in meta-analysis. BMJ Open.

[22] Higgins JPT, Thompson SG, Deeks JJ, Altman DG (2003). Measuring inconsistency in meta-analyses. BMJ.

[23] Rodrigues-Silva F, Santos U de P, Saldiva PHN, Amato-Lourenço LF, Miraglia SGEK. Health Risks and Economic Costs of Absenteeism Due to Air Pollution in São Paulo, Brazil. Aerosol Air Qual Res. Taiwan Association for Aerosol Research; 2012;12:826–33.

[24] Hales NM, Barton CC, Ransom MR, Allen RT, Pope CA (2016). A Quasi-Experimental Analysis of Elementary School Absences and Fine Particulate Air Pollution. Medicine (Baltimore).

[25] Marcon A, Pesce G, Girardi P, Marchetti P, Blengio G, de Zolt SS (2014). Association between PM10 concentrations and school absences in proximity of a cement plant in northern Italy. Int J Hyg Environ Health.

[26] Samoli E, Dimakopoulou K, Evangelopoulos D, Rodopoulou S, Karakatsani A, Veneti L (2017). Is daily exposure to ozone associated with respiratory morbidity and lung function in a representative sample of schoolchildren? Results from a panel study in Greece. J Expo Sci Environ Epidemiol.

[27] Bruyneel L, Kestens W, Alberty M, Karakaya G, Van Woensel R, Horemans C (2022). Short-Term exposure to ambient air pollution and onset of work incapacity related to mental health conditions. Environ Int.

[28] Willers SM, Eriksson C, Gidhagen L, Nilsson ME, Pershagen G, Bellander T (2013). Fine and coarse particulate air pollution in relation to respiratory health in Sweden. Eur Respir J.

[29] Gilliland FD, Berhane K, Rappaport EB, Thomas DC, Avol E, Gauderman WJ (2001). The effects of ambient air pollution on school absenteeism due to respiratory illnesses. Epidemiology.

[30] Ostro BD, Rothschild S (1989). Air pollution and acute respiratory morbidity: an observational study of multiple pollutants. Environ Res.

[31] Rondeau V, Berhane K, Thomas DC (2005). A three-level model for binary time-series data: the effects of air pollution on school absences in the Southern California Children’s Health Study. Stat Med.

[32] Chen S, Hong XU, Weiyan LIU, Shanshan XU, Ye LYU, Wenhui Z. The influence of air pollution on the health of primary school students. zgxxws. 中国学校卫生; 2021;42:1560–1563, 1567.

[33] Wu Y, Zhang T, Wang Y, Wei J, Huang L, Yang J, et al. Spatial heterogeneity in health risks of illness-related absenteeism associated with PM2.5 exposure for elementary students. Environ Res. 2022;212:113473. 10.1016/j.envres.2022.113473 35609651

[34] Yang, Huijian XIE, Wenpeng W, Yongyong XI, Cun QIN, Yunbiao H, et al. Air pollution and elementary school students’ absenteeism caused by respiratory symptoms and diseases among primary school students. Chinese Journal of School Health [Internet]. Chinese Journal of School Health; 2019 [cited 2022 Aug 25]; Available from: https://www.scienceopen.com/document?vid=1f769bd6-fe00-4210-818f-4b60805c75ec.

[35] Zhang Y, Cui L, Xu D, He MZ, Zhou J, Han L, et al. The association of ambient PM2.5 with school absence and symptoms in schoolchildren: a panel study. Pediatr Res. 2018;84:28–33. 10.1038/s41390-018-0004-1 PMC6581566 29795198

[36] Watanabe M, Noma H, Kurai J, Kato K, Sano H (2021). Association with Ambient Air Pollutants and School Absence Due to Sickness in Schoolchildren: A Case-Crossover Study in a Provincial Town of Japan. Int J Environ Res Public Health.

[37] Park H, Lee B, Ha E-H, Lee J-T, Kim H, Hong Y-C (2002). Association of air pollution with school absenteeism due to illness. Arch Pediatr Adolesc Med.

[38] Hansen AC, Selte HK (2000). Air pollution and sick-leaves A case study using air pollution data from Oslo. Environmental and Resource Economics Netherlands.

[39] Samakovlis E, Huhtala A, Bellander T, Svartengren M (2005). Valuing health effects of air pollution—Focus on concentration-response functions. J Urban Econ.

[40] Adams PF, Hendershot GE, Marano MA, Centers for Disease Control and Prevention/National Center for Health Statistics. Current estimates from the National Health Interview Survey, 1996. Vital Health Stat 10. 1999;1–203. 15782448

[41] Ostro BD (1983). The effects of air pollution on work loss and morbidity. J Environ Econ Manag.

[42] Alderson MR (1967). Data on sickness absence in some recent publications of the Ministry of Pensions and National Insurance. Br J Prev Soc Med.

[43] Bury IB (1970). A study of the effects of air pollution on children. J Sch Health.

[44] Künzli N, Kaiser R, Medina S, Studnicka M, Chanel O, Filliger P (2000). Public-health impact of outdoor and traffic-related air pollution: a European assessment. Lancet.

[45] Ostro BD (1990). Associations between morbidity and alternative measures of particulate matter. Risk Anal.

[46] Ostro BD, Tran H, Levy JI (2006). The health benefits of reduced tropospheric ozone in California. J Air Waste Manag Assoc.

[47] Castro A, Künzli N, Götschi T (2017). Health benefits of a reduction of PM10 and NO2 exposure after implementing a clean air plan in the Agglomeration Lausanne-Morges. Int J Hyg Environ Health.

[48] Malmqvist E, Lisberg Jensen E, Westerberg K, Stroh E, Rittner R, Gustafsson S (2018). Estimated health benefits of exhaust free transport in the city of Malmö. Southern Sweden Environ Int.

[49] Environmental noise guidelines for the European Region [Internet]. 2022 [cited 2022 Aug 25]. Available from: https://www.who.int/europe/publications/i/item/9789289053563.

[50] Lu C, Norbäck D, Zhang Y, Li B, Zhao Z, Huang C, et al. Common cold among young adults in China without a history of asthma or allergic rhinitis - associations with warmer climate zone, dampness and mould at home, and outdoor PM10 and PM2.5. Sci Total Environ. 2020;749:141580. 10.1016/j.scitotenv.2020.141580 32841860

[51] Yee J, Cho YA, Yoo HJ, Yun H, Gwak HS (2021). Short-term exposure to air pollution and hospital admission for pneumonia: a systematic review and meta-analysis. Environ Health.

[52] Orellano P, Quaranta N, Reynoso J, Balbi B, Vasquez J (2017). Effect of outdoor air pollution on asthma exacerbations in children and adults: Systematic review and multilevel meta-analysis. PLoS ONE.

[53] Zheng X-Y, Orellano P, Lin H-L, Jiang M, Guan W-J (2021). Short-term exposure to ozone, nitrogen dioxide, and sulphur dioxide and emergency department visits and hospital admissions due to asthma: A systematic review and meta-analysis. Environ Int.

[54] Bloemsma LD, Hoek G, Smit LAM (2016). Panel studies of air pollution in patients with COPD: Systematic review and meta-analysis. Environ Res.

[55] Chen J, Hoek G (2020). Long-term exposure to PM and all-cause and cause-specific mortality: A systematic review and meta-analysis. Environ Int.

[56] Song X, Wang S, Hu Y, Yue M, Zhang T, Liu Y (2017). Impact of ambient temperature on morbidity and mortality: An overview of reviews. Sci Total Environ.

[57] Analitis A, De’ Donato F, Scortichini M, Lanki T, Basagana X, Ballester F, et al. Synergistic Effects of Ambient Temperature and Air Pollution on Health in Europe: Results from the PHASE Project. Int J Environ Res Public Health. 2018;15:E1856. 10.3390/ijerph15091856 PMC6163671 30154318

[58] Janes H, Sheppard L, Lumley T (2005). Case-crossover analyses of air pollution exposure data: referent selection strategies and their implications for bias. Epidemiology.

[59] Brauer M, Brumm J, Vedal S, Petkau AJ (2002). Exposure misclassification and threshold concentrations in time series analyses of air pollution health effects. Risk Anal.

[60] Sterne JAC, Sutton AJ, Ioannidis JPA, Terrin N, Jones DR, Lau J (2011). Recommendations for examining and interpreting funnel plot asymmetry in meta-analyses of randomised controlled trials. BMJ.

[61] Rothstein HR, Sutton AJ, Borenstein M. Publication Bias in Meta-Analysis: Prevention, Assessment and Adjustments. Chichester: Wiley; 2005.

[62] Teculescu D, Pham QT, Aubry C, Chau N, Viaggi MN, Henquel JC, et al. [Respiratory health of children and atmospheric pollution. II. Ventilatory function]. Rev Mal Respir. 1989;6:221–8. 2740586

[63] Auermann E, Bigl S, Hajduk F, Meyer R (1992). Lippmann R [Effect of air pollutants on the health of the Annaberg district population with special reference to acute respiratory tract diseases]. Kinderarztl Prax.

[64] Krzyzanowski M, Dora C, Bruce N (2011). Improvement of air quality in low-income countries. Lancet.

