# Supplementary material for: Effects of air pollution on restricted activity days: systematic review and meta-analysis
Source: Environ Health. 2023 Mar 30;22:31. doi: 10.1186/s12940-023-00979-8 (PMC10061989; doi:10.1186/s12940-023-00979-8)
Supplement: Supplementary file 1 — Additional file 1: Supplementary Table 1. Search strategy: the example of MEDLINE via PubMed. Supplementary Table 2. Influential analysis for PM10 and work-loss/school-loss days. Supplementary Table 3. Influential analysis for PM2.5 and school-loss days. Supplementary Figure 1. Forest plot of one cross-sectional study (6 effect sizes) evaluating the association between PM2.5 and restricted activity days. Relative risks for a 10 µg/m3 increase in PM2.5 level. Supplementary Figure 2. Forest plot of one cross-sectional study (6 effect sizes) evaluating the association between PM2.5 and respiratory-related restricted activity days. Relative risks for a 10 µg/m3 increase in PM2.5 level. Supplementary Figure 3. Forest plot of one cross-sectional study (6 effect sizes) evaluating the association between PM2.5 and minor restricted activity days. Relative risks for a 10 µg/m3 increase in PM2.5 level. Supplementary Figure 3. Forest plot of one cross-sectional study (6effect sizes) evaluating the association between PM2.5 and minor restricted activity days. Relative risks for a 10 µg/m3 increase in PM2.5 level. Supplementary Figure 4. Forest plot of one cross-sectional study (6 effect sizes) evaluating the association between PM2.5 and work-loss days. Relative risks for a 10 µg/m3 increase in PM2.5 level. Supplementary Table 4. Influential analysis for NO2 and work-loss/school-loss days. Supplementary Figure 5. Forest plot of one cross-sectional study (6 effect sizes) evaluating the association between O3 and respiratory-related restricted activity days. Relative risks for a 10 µg/m3 increase in O3 level. Supplementary Figure 6. Forest plot of one cross-sectional study (6 effect sizes) evaluating the association between O3 and minor restricted activity days. Relative risks for a 10 µg/m3 increase in O3 level. [file 12940_2023_979_MOESM1_ESM.docx]

**Additional File 1.**

**Supplementary Table 1.** Search strategy: the example of MEDLINE via PubMed.

|  | **Search timeline**: Up to 13.Jun.2022 | **Abstracts** |
| --- | --- | --- |
| **#3** | **Search**: #1 AND #2 | 586 |
| **#2** | **Search**: ("activity days"[TIAB] OR "restricted activity days"[TIAB] OR "minor restricted activity days"[TIAB] OR "RAD"[TIAB] OR "RADs"[TIAB] OR "MRAD"[TIAB] OR "MRADs"[TIAB] OR "work loss"[TIAB] OR "work-loss"[TIAB] OR "lost work"[TIAB] OR "sick leaves"[TIAB] OR "absenteeism"[TIAB] OR "absenteeism"[MH] OR "nonattendance"[TIAB] OR "non-attendance"[TIAB] OR "attendance"[TIAB] OR "school loss"[TIAB] OR "school-loss"[TIAB] OR "bed days"[TIAB] OR "bed-days"[TIAB]) | 68,057 |
| **#1** | **Search**: ("particulate matter"[TIAB] OR "particulate air"[TIAB] OR "suspended particles"[TIAB] OR "coarse particles"[TIAB] OR "respirable particles"[TIAB] OR "fine particles"[TIAB] OR "inhalable particles"[TIAB] OR "SPM"[TIAB] OR "TSP"[TIAB] OR "PM10"[TIAB] OR "PM2.5"[TIAB] OR "particulate matter"[MH] OR "NO2"[TIAB] OR "nitrogen dioxide"[MH] OR "nitrogen oxides"[MH] OR "NOx"[TIAB] OR "ozone"[TIAB] OR "O3"[TIAB] OR "ozone"[MH] OR "air pollution"[TIAB] OR "air pollution"[MH] OR "air pollutants"[TIAB] OR "air pollutants"[MH]) | 335,423 |

**Supplementary Table 2.** Influential analysis for PM_10_ and work-loss/school-loss days.

| **Study excluded** | **RR (95%CI)** | **I2** |
| --- | --- | --- |
| Gilliland, 2001 | 1.0173 (1.0036-1.0313) | 74% |
| Hansen, 2000 | 1.0149 (1.0017-1.0283) | 69% |
| Marcon, 2014 | 1.0157 (0.9995-1.0323) | 70% |
| Park, 2002 | 1.0201 (0.9982-1.0426) | 74% |
| Rondeau, 2005 | 1.0191 (1.0058-1.0326) | 71% |
| Yang, 2019 | 1.0235 (1.0092-1.0379) | 51% |
| Chen, 2021 | 1.0132 (0.9998-1.0268) | 66% |

PM_10_, particulate matter with aerodynamic diameters less or equal than 10 μm; RR, pooled relative risk, calculated for a 10 μg/m^3^ increase in the pollutant level; 95% CI, 95% confidence interval; I2, I-Squared values.

**Supplementary Table 3.** Influential analysis for PM_2.5_ and school-loss days.

| **Study excluded** | **RR (95%CI)** | **I2** |
| --- | --- | --- |
| Hales, 2016 (Alpine) | 1.0170 (1.0036-1.0306) | 98% |
| Hales, 2016 (Provo) | 1.0166 (1.0031-1.0304) | 99% |
| Hales, 2016 (Salt Lake) | 1.0190 (1.0127-1.0253) | 92% |
| Watanabe, 2021 | 1.0145 (1.0030-1.0262) | 99% |
| Wu, 2022 | 1.0196 (1.0053-1.0341) | 99% |
| Yang, 2019 | 1.0178 (1.0052-1.0307) | 99% |
| Zhang, 2018 | 1.0166 (1.0050-1.0283) | 99% |
| Chen, 2021 | 1.0168 (1.0041-1.0297) | 99% |

PM_2.5_, particulate matter with aerodynamic diameters less or equal than 10 μm; RR, pooled relative risk, calculated for a 10 μg/m^3^ increase in the pollutant level; 95% CI, 95% confidence interval; I2, I-Squared values.

**Supplementary Figure 1.** Forest plot of one cross-sectional study (6 effect sizes) evaluating the association between PM_2.5_ and restricted activity days. Relative risks for a 10 µg/m^3^ increase in PM_2.5_ level.

**
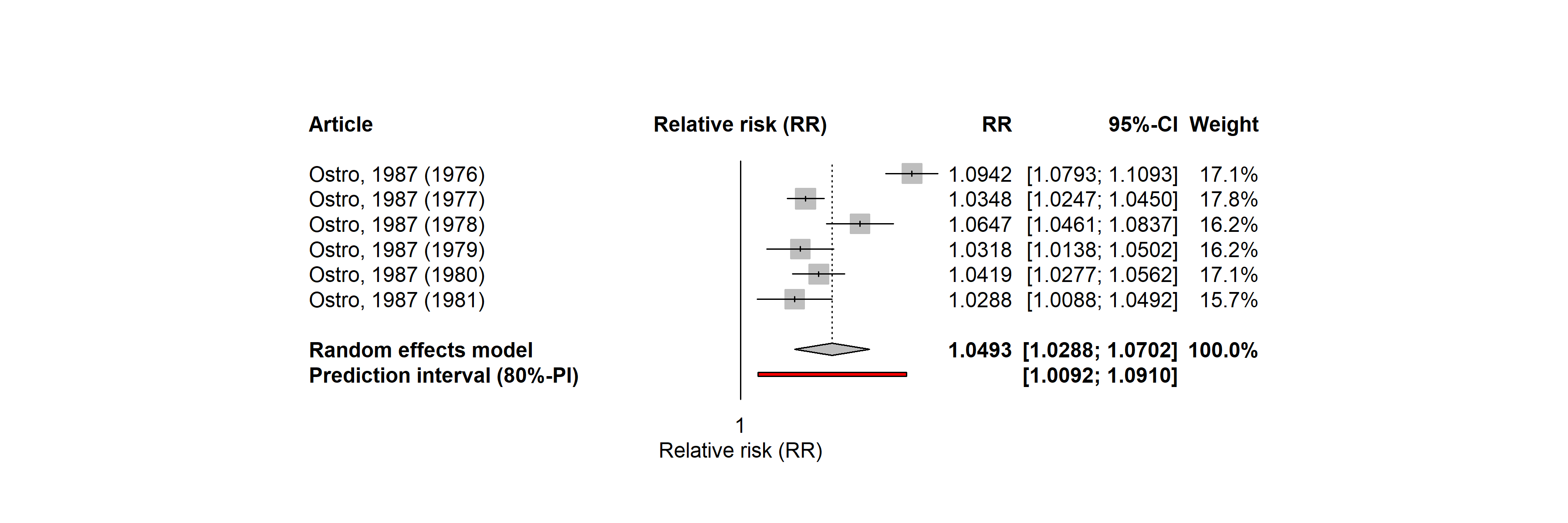
**

**Supplementary Figure 2.** Forest plot of one cross-sectional study (6 effect sizes) evaluating the association between PM_2.5_ and respiratory-related restricted activity days. Relative risks for a 10 µg/m^3^ increase in PM_2.5_ level.

**
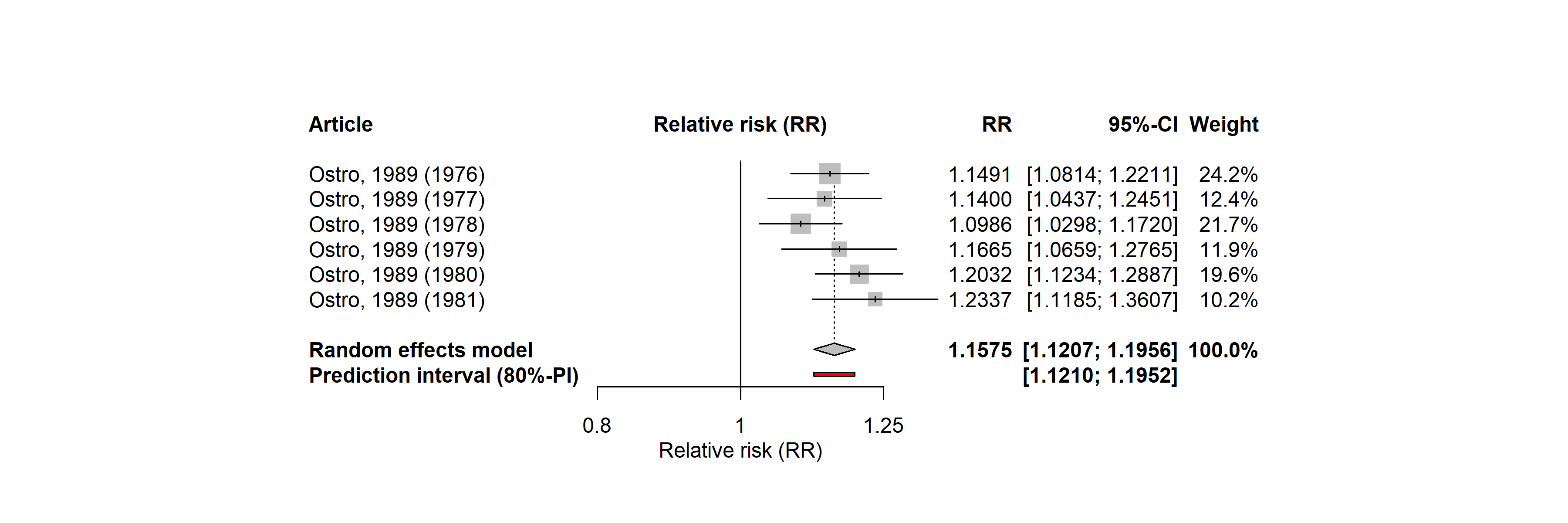
**

**Supplementary Figure 3.** Forest plot of one cross-sectional study (6 effect sizes) evaluating the association between PM_2.5_ and minor restricted activity days. Relative risks for a 10 µg/m^3^ increase in PM_2.5_ level.

**
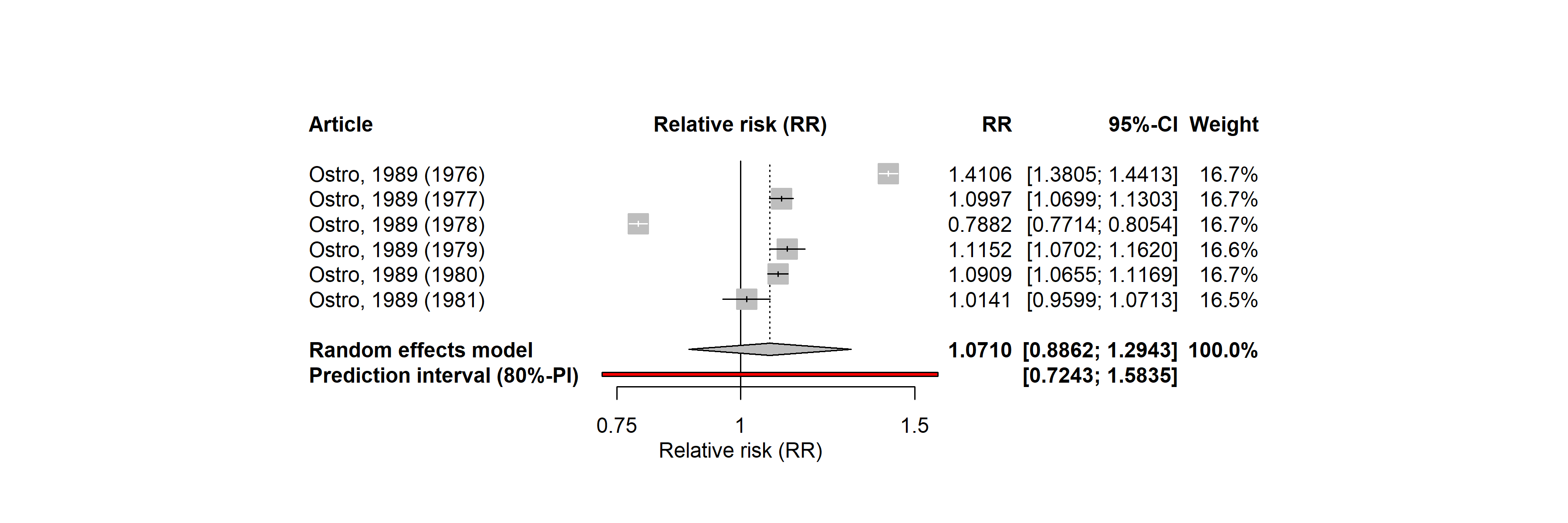
**

**Supplementary Figure 4.** Forest plot of one cross-sectional study (6 effect sizes) evaluating the association between PM_2.5_ and work-loss days. Relative risks for a 10 µg/m^3^ increase in PM_2.5_ level.

**
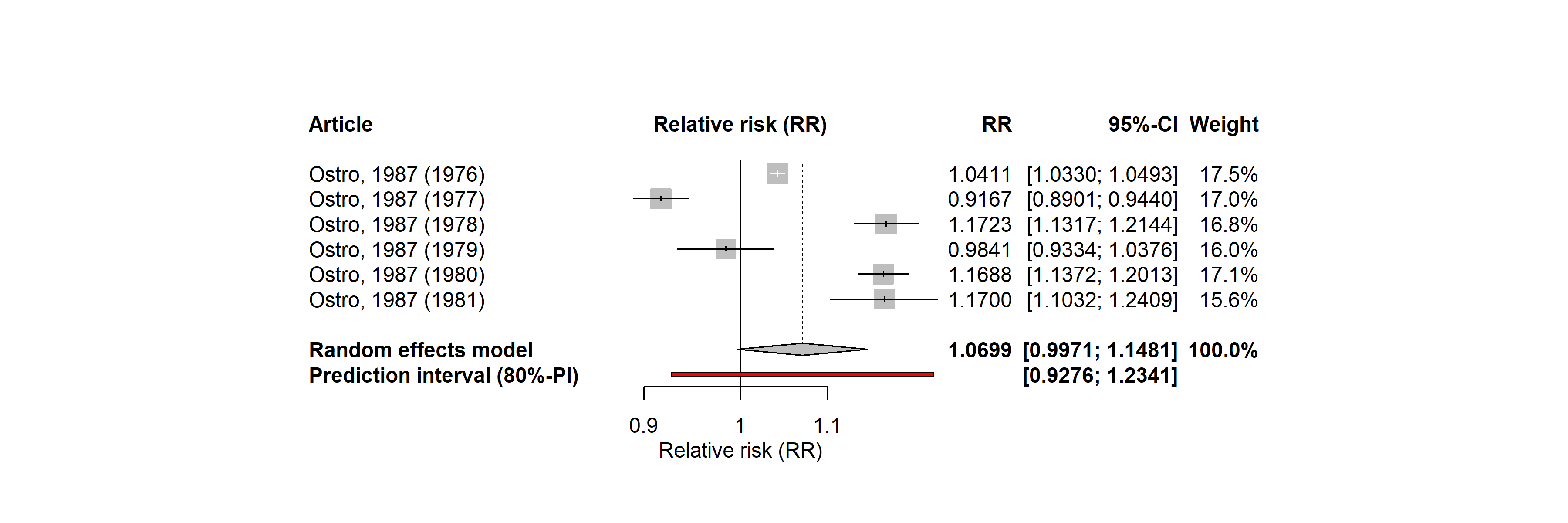
**

**Supplementary Table 4.** Influential analysis for NO_2_ and work-loss/school-loss days.

| **Study excluded** | **RR (95%CI)** | **I2** |
| --- | --- | --- |
| Gilliland, 2001 | 1.0076 (0.9913-1.0242) | 80% |
| Hansen, 2000 | 1.0085 (0.9909-1.0264) | 80% |
| Park, 2002 | 1.0074 (0.9832-1.0321) | 80% |
| Rondeau, 2005 | 1.0147 (1.0012-1.0284) | 59% |
| Watanabe, 2021 | 1.0030 (0.9863-1.0199) | 77% |
| Yang, 2019 | 1.0091 (0.9903-1.0283) | 80% |
| Chen, 2021 | 1.0021 (0.9854-1.0191) | 68% |

NO_2_, nitrogen dioxide; RR, pooled relative risk, calculated for a 10 μg/m^3^ increase in the pollutant level; 95% CI, 95% confidence interval; I2, I-Square values.

**Supplementary Table 5.** Influential analysis for O_3_ and school-loss days.

| **Study excluded** | **RR (95%CI)** | **I2** |
| --- | --- | --- |
| Gilliland, 2001 | 0.9944 (0.9543-1.0362) | 88% |
| Park, 2002 | 1.0095 (0.9147-1.1141) | 84% |
| Rondeau, 2005 | 1.0274 (0.9442-1.1180) | 80% |
| Samoli, 2017 | 1.0254 (0.9824-1.0703) | 90% |

O_3_, ozone; RR, pooled relative risk, calculated for a 10 μg/m^3^ increase in the pollutant level; 95% CI, 95% confidence interval; I2, I-Square values.

**Supplementary Figure 5.** Forest plot of one cross-sectional study (6 effect sizes) evaluating the association between O_3_ and respiratory-related restricted activity days. Relative risks for a 10 µg/m^3^ increase in O_3_ level.

**
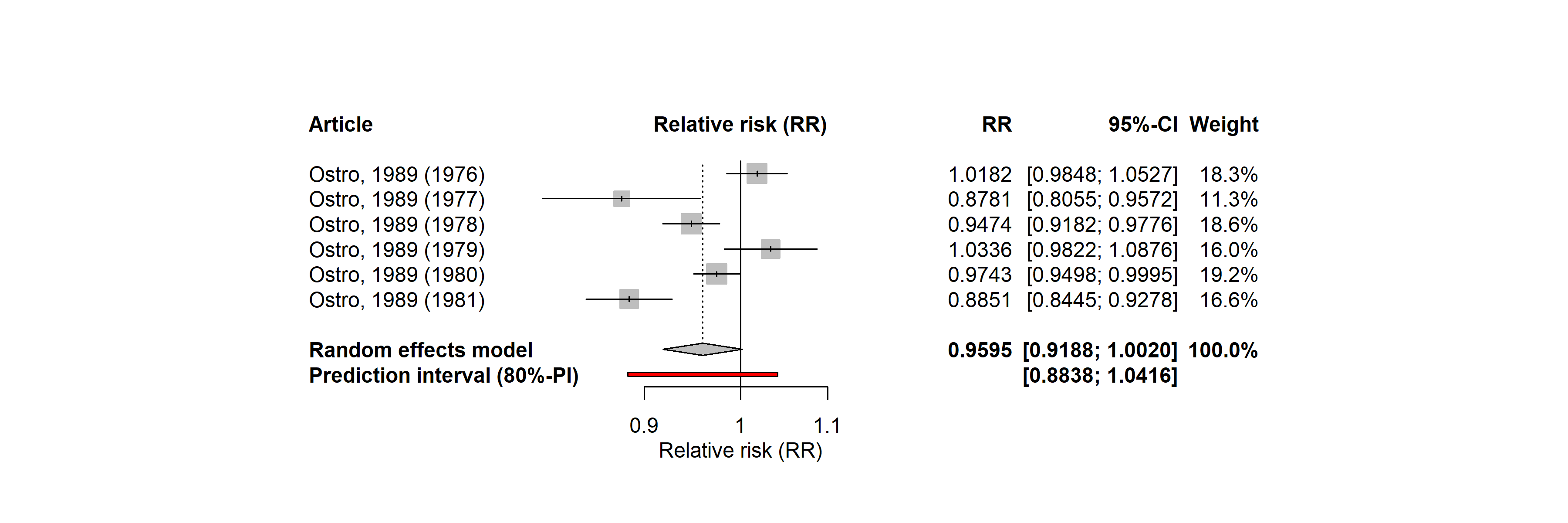
**

**Supplementary Figure 6.** Forest plot of one cross-sectional study (6 effect sizes) evaluating the association between O_3_ and minor restricted activity days. Relative risks for a 10 µg/m^3^ increase in O_3_ level.

**
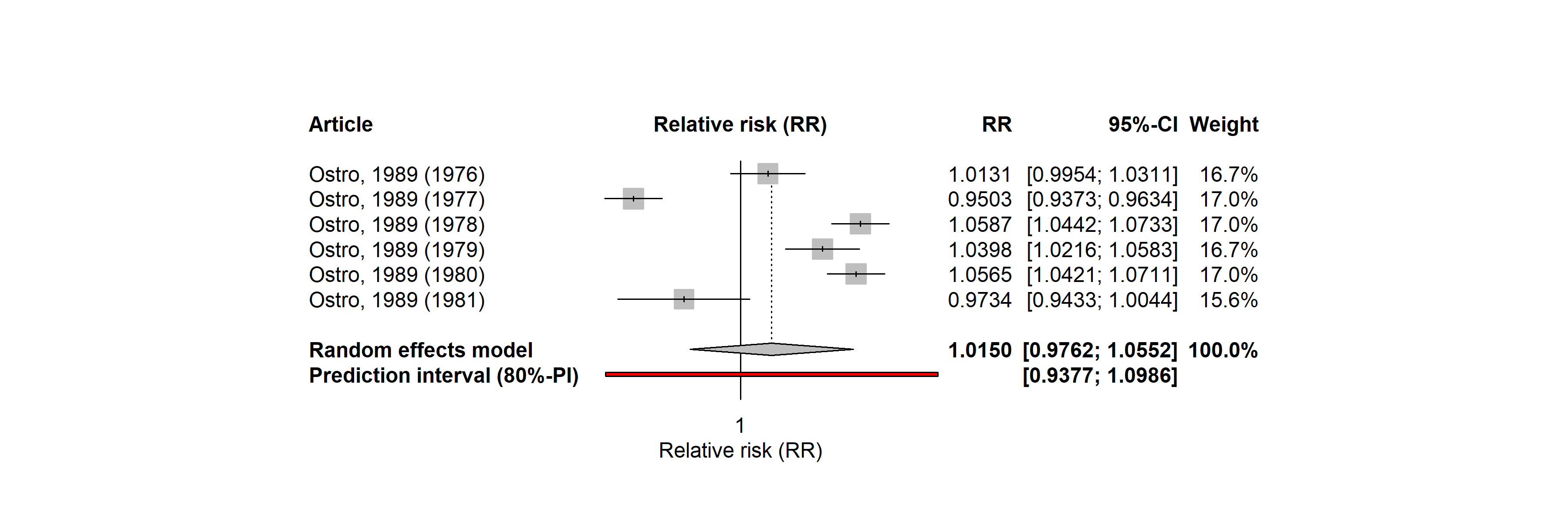
**
